# Anti-latent TGFβ binding protein 4 antibody improves muscle function and reduces muscle fibrosis in muscular dystrophy

**DOI:** 10.1126/scitranslmed.abf0376

**Published:** 2021-09-08

**Authors:** Alexis R. Demonbreun, Katherine S. Fallon, Claire C. Oosterbaan, Lauren A. Vaught, Nina L. Reiser, Elena Bogdanovic, Matthew P. Velez, Isabella M. Salamone, Patrick G.T. Page, Michele Hadhazy, Mattia Quattrocelli, David Y. Barefield, Lauren D. Wood, J. Patrick Gonzalez, Carl Morris, Elizabeth M. McNally

**Affiliations:** 1 Center for Genetic Medicine, Northwestern University, Chicago, IL 60611, USA.; 2 Department of Pharmacology, Northwestern University, Chicago, IL 60611, USA.; 3 Solid Biosciences, Cambridge, MA 02139, USA.

## Abstract

Duchenne muscular dystrophy, like other muscular dystrophies, is a progressive disorder hallmarked by muscle degeneration, inflammation, and fibrosis. Latent transforming growth factor β (TGFβ) binding protein 4 (LTBP4) is an extracellular matrix protein found in muscle. LTBP4 sequesters and inhibits a precursor form of TGFβ. LTBP4 was originally identified from a genome-wide search for genetic modifiers of muscular dystrophy in mice, where there are two different alleles. The protective form of LTBP4, which contains an insertion of 12 amino acids in the protein’s hinge region, was linked to increased sequestration of latent TGFβ, enhanced muscle membrane stability, and reduced muscle fibrosis. The deleterious form of LTBP4 protein, lacking 12 amino acids, was more susceptible to proteolysis and promoted release of latent TGF-β, and together, these data underscored the functional role of LTBP4’s hinge. Here, we generated a monoclonal human anti-LTBP4 antibody directed toward LTBP4’s hinge region. In vitro, anti-LTBP4 bound LTBP4 protein and reduced LTBP4 proteolytic cleavage. In isolated myofibers, the LTBP4 antibody stabilized the sarcolemma from injury. In vivo, anti-LTBP4 treatment of dystrophic mice protected muscle against force loss induced by eccentric contraction. Anti-LTBP4 treatment also reduced muscle fibrosis and enhanced muscle force production, including in the diaphragm muscle, where respiratory function was improved. Moreover, the anti-LTBP4 in combination with prednisone, a standard of care for Duchenne muscular dystrophy, further enhanced muscle function and protected against injury in *mdx* mice. These data demonstrate the potential of anti-LTBP4 antibodies to treat muscular dystrophy.

## INTRODUCTION

Duchenne muscular dystrophy (DMD) is caused by loss of the membrane-associated protein dystrophin (1), and mutations in genes encoding dystrophin-associated proteins such as the sarcoglycans elicit a similar phenotype (2, 3). These disorders are characterized by fragile muscle membranes that are highly prone to injury and disruption. Repetitive injury to myofibers produces extensive muscle degeneration, increased tissue inflammation, and, ultimately, replacement by fibrosis and fatty infiltrate. Clinically, over time, this process causes loss of ambulation, weakened breathing, and impaired heart function.

Latent transforming growth factor β (TGFβ) binding protein 4 (LTBP4) is a 160-kDa matrix-embedded protein that is a member of the fibrillin super gene family. LTBPs bind to the latent forms of TGFβ, holding these proteins inactive and unavailable to cell surface receptors (4). LTBP4 binds to latent TGFβ1, TGFβ2, and TGFβ3, as well as the highly related TGFβ family member myostatin (growth and differentiation factor 8) (5, 6). *Ltbp4* was originally identified as a genetic modifier of mouse muscular dystrophy from an unbiased genome-wide search for modifiers (7). There are two major alleles found in mice, and most mouse strains have the protective allele. The protective allele of *Ltbp4* encodes an in-frame insertion of 36 base pairs into exon 12, which encodes the “hinge” region of LTBP4 protein. The strongest *Ltbp4* genomic signals were linked to sarcolemmal stability, measured as membrane leak, and a reduction in fibrosis (7, 8). The protective mouse *Ltbp*4 allele encodes a protein with reduced susceptibility to proteolysis and increased sequestration of TGFβ ligands, reducing their deleterious signaling cascades on dystrophic muscle pathology (5, 7, 9). LTBP4 is broadly expressed but highly increased after muscle injury (9). The deleterious mouse *Ltbp4* allele is found in the DBA/2J strain and is characterized by a shorter hinge region. Human LTBP4 has an even shorter hinge region than found in the DBA/2J strain, further increasing the susceptibility of LTBP4 to protease cleavage and TGFβ release (9). Transgenic overexpression of the protective LTBP4 isoform in dystrophic mice was shown to reduce fibrosis, increase muscle mass, and enhance muscle strength (5).

Human genetic data also support a role for *LTBP4* in human muscular dystrophy. Patients with DMD with specific *LTBP4* single-nucleotide polymorphisms have prolonged ambulation (10). This result was replicated in independent cohorts of human DMD subjects, illustrating the strong modifying effect of this pathway (11, 12). The human protective LTBP4 polymorphisms encode a protein that results in tighter binding to latent TGFβ, leading to reduced TGFβ signaling (6).

We hypothesized that blocking LTBP4’s hinge region would stabilize the protective conformation of LTBP4. Here, we describe the development and testing of monoclonal human and mouse antibodies directed against the human LTBP4 hinge region. The anti-LTBP4 antibody localized within muscle in the expected costameric pattern, and a single injection was detected in mice in vivo for at least 21 days. Using mouse models of DMD, we showed that short-term anti-LTBP4 antibody exposure improved sarcolemmal stability and reduced the susceptibility to eccentric contraction (ECC)–induced force loss. Long-term 24-week administration of anti-LTBP4 antibody improved muscle performance and reduced muscle fibrosis, including in the diaphragm muscle, one of the most adversely affected muscles in DMD and its mouse models. Together, these data demonstrate that stabilizing LTBP4 through anti-LTBP4 antibody administration is an effective strategy to reduce muscle fibrosis and increase muscle performance in DMD.

## RESULTS

### A monoclonal antibody to LTBP4 recognizes human and mouse LTBP4 protein

**Fig. 1.**
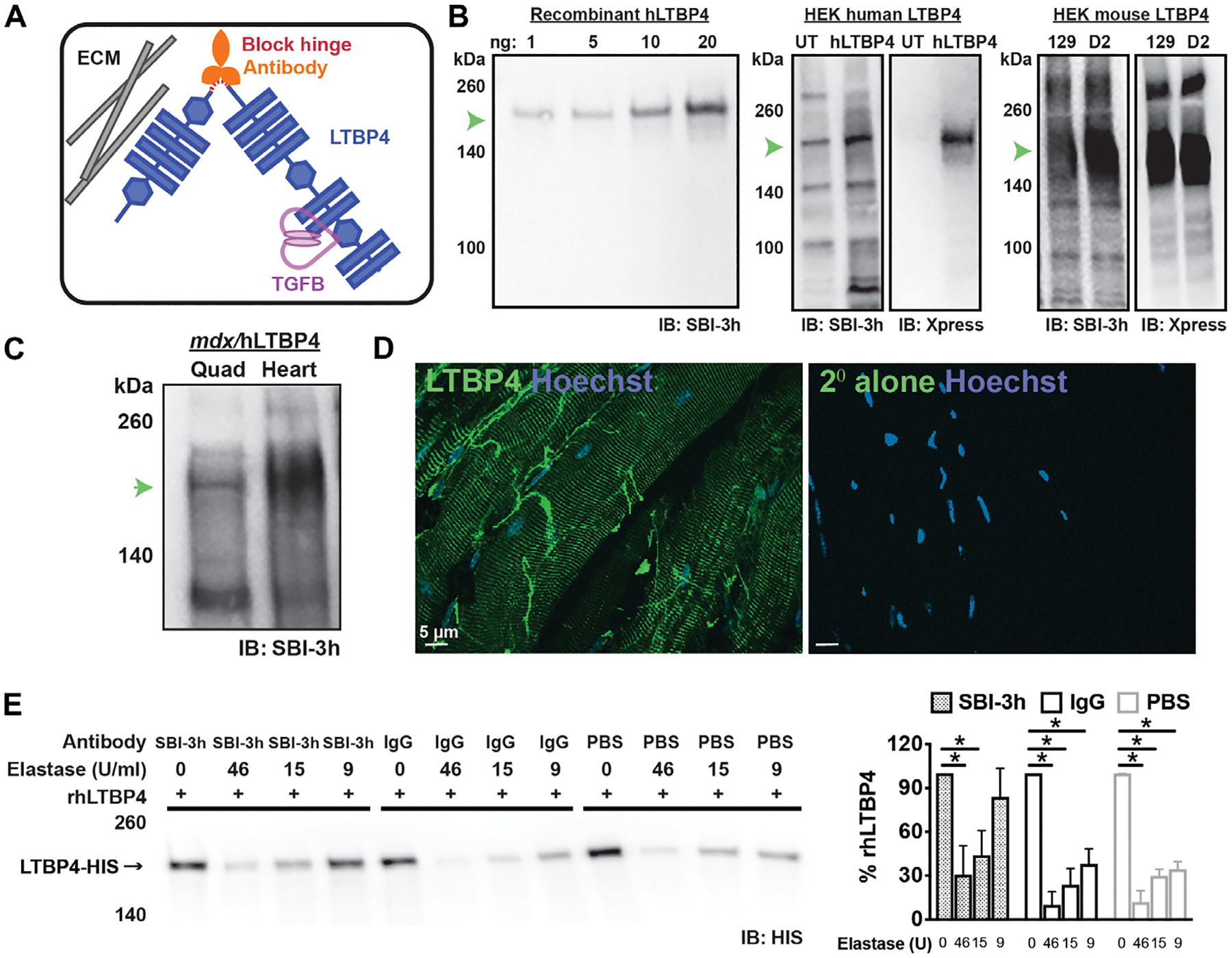
Targeting the LTBP4 hinge region using a monoclonal antibody. (**A**) The hinge region of LTBP4 is cleaved by serum proteases. After cleavage, latent TGFβ is released where it can be further activated; the anti-LTBP4 antibody protects LTBP4 from cleavage. ECM, extracellular matrix. (**B**) SBI-3h demonstrated high affinity and specificity to recombinant human LTBP4 protein and to human LTBP4 overepxressed in HEK cells. The overexpressed LTBP4 was also detected by its Xpress epitope tag. Endogenously expressed LTBP4 was also present in HEK cells [detected in the (UT, untransfected) lane]. SBI-3h also recognized the two different forms of mouse LTBP4 protein encoded by the 129T2/SvEmsJ (129) and DBA2/J (D2) substrains. IB, immunoblotting. (**C**) SBI-3h recognized LTBP4 protein found in lysates generated from *mdx*/hLTBP4 mouse muscle (quadriceps) and heart. (**D**) Immunofluorescence microscopic imaging of unfixed isolated myofibers showed SBI-3h (green) localized in a striated pattern. Staining of nonfixed fibers reflects LTBP4 on the external cell surface in a costameric pattern. Hoechst (blue) marks nuclei. Scale bar, 5 μm. (**E**) Recombinant human LTBP4 (rhLTBP4) was incubated with the high-affinity anti-LTBP4 antibody (SBI-3h), IgG control antibody, or PBS and then subjected to elastase exposure with varying concentrations of elastase. Incubation with anti-LTBP4 antibody protected against LTBP4 proteolysis seen as an increased percentage of full-length LTBP4 at 9 U of elastase. *P < 0.05 by one-way ANOVA (*n* = 4).

We investigated the translational potential of an antibody-based strategy to stabilize the hinge of LTBP4 because prior work established the hinge as a critical component of latent TGFβ stability (Fig. 1A) (7, 9). To generate an antibody specific for an epitope within the LTBP4 hinge, screening was performed using peptides corresponding to the conserved amino acid residues in the proline-arginine (PR)–enriched hinge region. This region includes a repetitive PRPRP sequence shared across human and mouse isoforms.

A human monoclonal anti-LTPB4 antibody, termed SBI-3h, recognized purified recombinant human LTBP4 protein on immunoblotting, at the expected 160-kDa molecular weight, over a range of protein concentrations, from 1 to 20 ng (Fig. 1B, left). SBI-3h detected LTBP4 protein in lysates from human embryonic kidney 293 (HEK293) cells overexpressing human LTBP4 protein (Fig. 1B, middle). SBI-3h also detected endogenous LTBP4 protein in untransfected HEK293 cells (Fig. 1B, middle). LTBP4 overexpression was confirmed using an antibody to the Xpress epitope tag present on the C terminus of the overexpressed LTBP4 protein (Fig. 1B, middle). Although a sequence corresponding to the human LTBP4 hinge was used to generate SBI-3h, this antibody also reacted with mouse LTBP4. Epitope-tagged constructs of either the protective *Ltbp4* or the deleterious *Ltbp4* alleles, differing at the hinge region, were expressed in HEK293 cells. SBI-3h recognized both murine isoforms (Fig. 1B, right). This cross-reactivity, in which SBI-3h recognizes human and mouse LTBP4 proteins, likely reflects the repetitive sequence found in the hinge region. We transferred the human variable region onto a mouse immunoglobulin G1 (IgG1) framework to create SBI-3m, and this resulted in LTBP4 recognition similar to SBI-3h (fig. S1).

We previously developed a mouse model of DMD that expressed human *LTBP4* in vivo from a bacterial artificial chromosome transgene under the endogenous *LTBP4* promoter (9). This mouse model has a more severe form of muscular dystrophy than the background-matched *mdx* mouse model because the human LTBP4 is more readily proteolyzed with increased TGFβ signaling. It is referred to as *mdx*/hLTBP4 and expresses a mixture of both human and mouse forms of LTBP4 protein. Lysates prepared from *mdx*/hLTBP4 quadriceps muscle and heart both showed expression of LTBP4 protein detected by SBI-3h (Fig. 1C). LTBP4 protein is localized in a costameric pattern on the outside of myofibers (5, 9). The SBI-3h antibody demonstrated a similar costameric pattern on isolated, nonfixed/nonpermeabilized isolated myofibers, consistent with its position on the exterior of myofibers (Fig. 1D). These data demonstrate that SBI-3h detects LTBP4 protein.

### SBI-3h protects LTBP4 against protease cleavage

We previously showed that LTBP4 protein was proteolyzed by the serine proteases plasmin or elastase at its hinge region (7, 9). We tested whether SBI-3h protected against elastase-induced cleavage. Full-length human recombinant LTBP4 was incubated across a range of elastase concentrations in the presence or absence of SBI-3h. Incubation with SBI-3h protected against elastase cleavage compared to a nonspecific IgG and no-antibody controls, as evidenced by increased amounts of full-length LTBP4 protein remaining after digestion (Fig. 1E).

### SBI-3h is detected 21 days after in vivo administration

The longevity and targeting of anti-LTBP4 antibody were evaluated in vivo. Mice were injected with the human antibody SBI-3h and then evaluated for the presence of human IgG after injection. *md*x/hLTBP4 mice were injected once intraperitoneally with SBI-3h (10 mg/kg) and then euthanized for analysis at 1, 7, 14, or 21 days after the single injection. As controls, *md*x/hLTBP4 was injected with phosphate-buffered saline (PBS). Myofibers were then isolated and immunostained with anti-human IgG conjugated to Alexa 488 to visualize the presence of SBI-3h. SBI-3h was detected by anti-human IgG–Alexa 488 and was visible at 1, 7, 14, and even 21 days after injection in a striated, costameric pattern, consistent with the known pattern of LTBP4 in muscle. There was no detectable anti-human IgG signal in the PBS-injected muscle (fig. S2). The costameric anti-human IgG signal was more apparent at days 7 and 14 compared to day 1 or 21. These data are consistent with SBI-3h binding LTBP4 in muscle and remain detectable for at least 21 days after a single intraperitoneal injection.

### Short-term SBI-3h dosing enhances muscle function in mdx mice

**Fig. 2.**
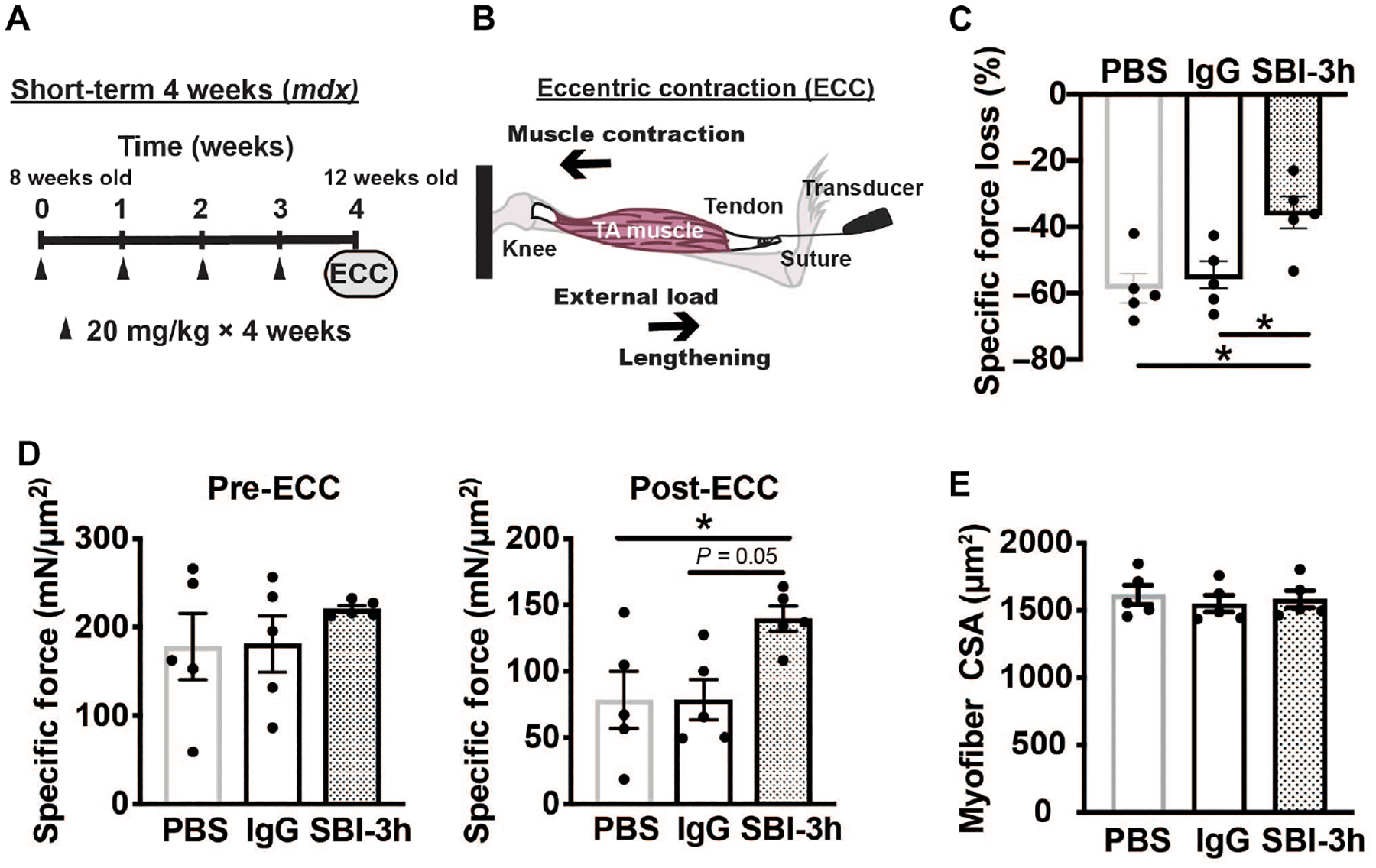
Short-term in vivo treatment with anti-LTBP4 human antibody enhanced muscle performance in dystrophic mice. (**A**) *mdx* male mice were injected intraperitoneally with either PBS, human IgG control antibody, or SBI-3h at 20 mg/kg once per week for 4 weeks starting at 8 weeks of age and completing at 12 weeks of age. Arrowheads indicate the timing of antibody dosing. (**B**) Muscle lengthening through application of external force during muscle contraction elicits eccentric contraction (ECC)–induced injury to *mdx* muscle. (**C**) Specific force loss after eccentric injury is a feature of *mdx* TA muscle after lengthening contractions (ECC) due to dystrophin loss. Treatment with SBI-3h, but not nonspecific IgG, reduced the amount of force loss in treated TA muscles, indicating protection against injury and preserved performance. (**D**) TA muscle–specific force measurements before eccentric injury and after eccentric injury in anti-LTBP4 (SBI-3h)–treated mice. (**E**) TA myofiber cross-sectional area measurements after treatment with antibody. * *P* < 0.05 by one-way ANOVA. *n* = 5 mice per group.

To evaluate the in vivo effects of anti-LTBP4 antibodies on dystrophic muscle, *mdx* mice were injected intraperitoneally with SBI-3h at a dosage of 20 mg/kg, once weekly, for four consecutive weeks (Fig. 2A). PBS and human IgG were administered as controls. Before sacrifice, at the 4-week time point, tibialis anterior (TA) muscle function was evaluated in situ using ECC as a model of muscle injury (Fig. 2B). The ECC protocol evaluates force production after lengthening contractions that injure dystrophin-deficient muscle resulting in force loss (13–15). Mice treated with SBI-3h had a decrease in the percent-specific force drop after in situ ECC-induced injury compared to PBS and human IgG controls (Fig. 2C). This decrease in force drop was the outcome of the combination of pre-ECC–specific force and post-ECC–specific force production in SBI-3h–treated mice (Fig. 2D). In this short-term dosing, administration of SBI-3h did not elicit observable changes in TA myofiber cross-sectional area (Fig. 2E), which indicated that antibody treatment likely exerted its effect through protection from injury rather than any promotion of growth during this short treatment. Thus, 4 weeks of injections with an anti-LTBP4 antibody was sufficient to alleviate susceptibility of dystrophic muscle to ECC-induced force loss.

### LTBP4 antibody targets muscle and reduces phospho-SMAD in mdx mice

**Fig. 3.**
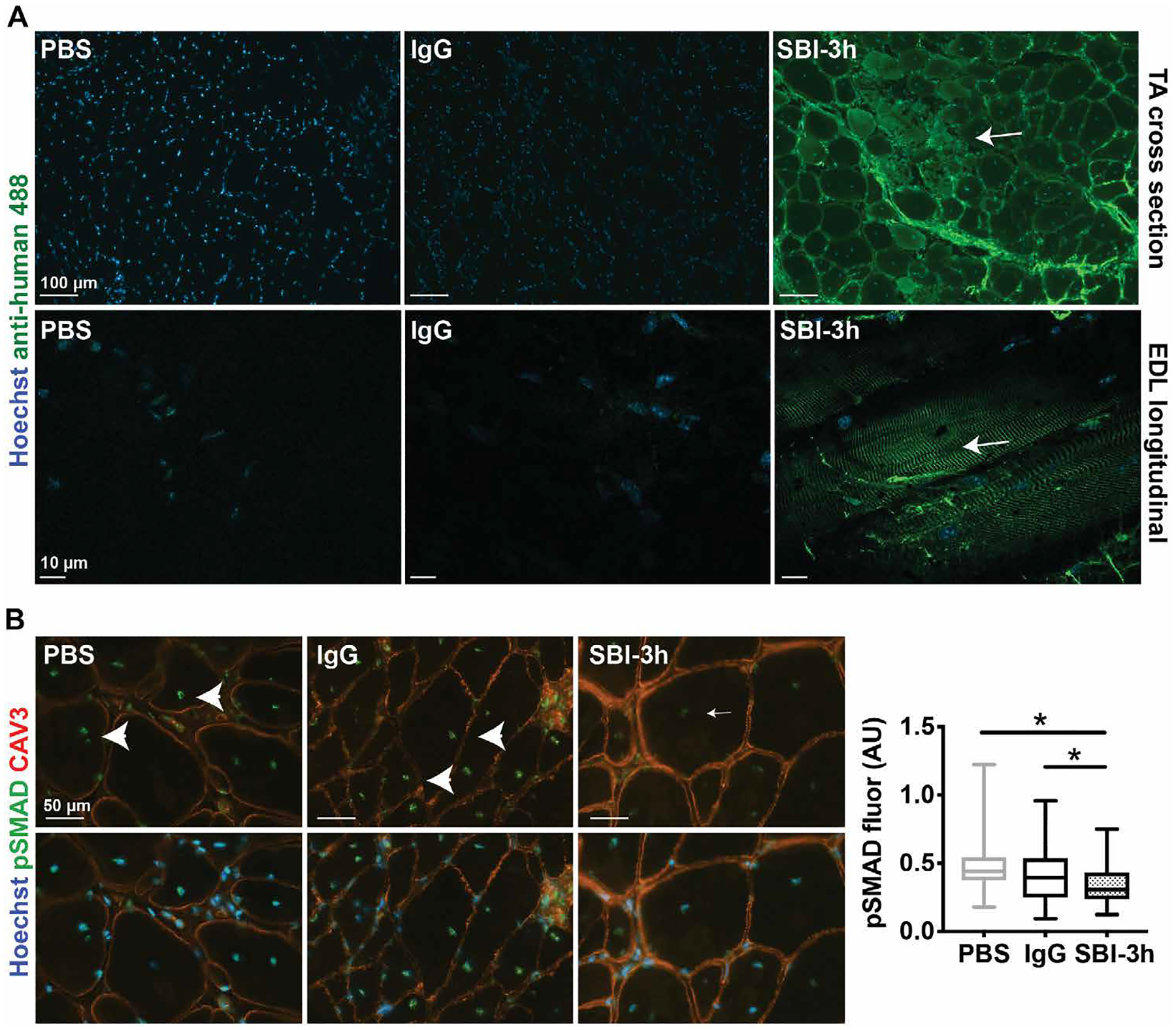
In vivo injected anti-LTBP4 human antibody localized to muscle and reduced pSMAD signaling in dystrophic mice. *mdx* male mice were injected intraperitoneally with either PBS, human IgG control antibody, or SBI-3h at 20 mg/kg once per week for 4 weeks starting at 8 weeks of age. (**A**) An anti-human secondary antibody (green) was used to detect the presence of SBI-3h in ECC-injured TA muscle in cross sections between and at the periphery of myofibers (top; scale bars, 100 μm) or in longitudinal sections at costameres of noninjured EDL muscle (bottom; scale bars, 10 μm). Hoechst dye (blue) marked nuclei. (**B**) Anti-pSMAD (green) immunofluorescence intensity was decreased (small arrow) in internal nuclei of TA muscle from SBI-3h–injected mice after eccentric injury compared to controls (arrowheads). Anti–caveolin 3 (red) was used as a membrane marker. Hoechst (blue) marked nuclei. Scale bars, 50 μm. *P < 0.05 by one-way ANOVA. *n* = 5 mice per group. AU, arbitrary units.

Using anti-human IgG, we demonstrated that SBI-3h was present in the TA muscles that showed resistance to ECC injury and was absent in control muscle (Fig. 3A, top). In addition, non-ECC–challenged extensor digitorum longus (EDL) muscles from the same mice were stained and showed costameric fluorescence signal only in mice dosed with SBI-3h (Fig. 3A, bottom). The presence of anti-human 488 fluorescence signal indicated the presence of human LTBP4 antibody SBI-3h within multiple muscle groups. LTBP4 regulates the bioavailability of TGFβ, modulating down-stream SMAD phosphorylation (16, 17). We evaluated the effects of SBI-3h administration on SMAD phosphorylation (pSMAD), focusing the analysis on internal myonuclei that are known to be pSMAD-positive after injury (9). Probing internal nuclei from ECC-challenged TA muscle for pSMAD immunofluorescence revealed a reduction in pSMAD in animals treated with SBI-3h as compared to PBS or human IgG control animals (Fig. 3B). These data show that after 4 weeks of treatment, anti-LTBP4 antibody properly localized in skeletal muscle and decreased pSMAD in myonuclei in vivo.

### SBI-3h protects against sarcolemmal injury

**Fig. 4.**
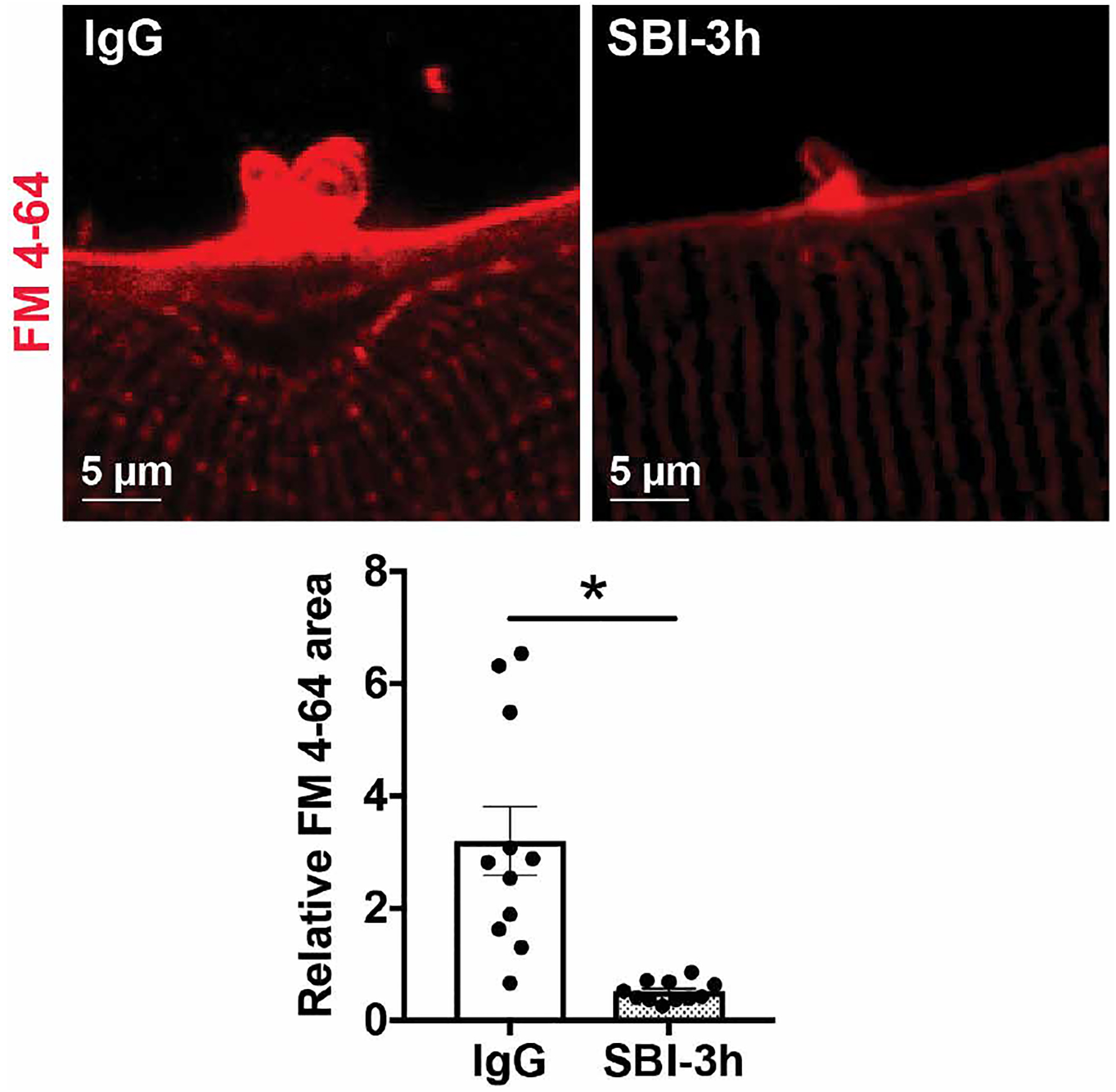
Pretreatment with the anti-LTBP4 human antibody protected myofibers from membrane injury. *mdx*/hLTBP4 mice express human LTBP4 from a transgene. Myofibers from these mice were isolated and pretreated with SBI-3h and then subjected to laser-induced membrane injury in the presence of FM 4–64 (red) to mark membrane damage. Myofibers treated with anti-LTBP4 antibody had reduced FM 4–64 fluorescence area compared to human IgG-treated control myofibers. Scale bars, 5 μm. *P < 0.05 by two-tailed *t* test. *n* ≥ 3 animals per treatment with multiple fibers per mouse.

The protective form of mouse LTBP4 was previously shown to modify the susceptibility of dystrophic myofibers to sarcolemmal injury in vitro and in vivo (18). To evaluate the effect of SBI-3h on sarcolemmal injury, *mdx*/hLTBP4 flexor digitorum brevis (FDB) myofibers were isolated and incubated with PBS, human IgG, or SBI-3h antibody for 2 hours. Subsequently, myofibers were subjected to laser-induced membrane injury in the presence of FM 4–64, a fluorescent dye that marks membrane injury (19, 20). FM 4–64 is a membrane-impermeable dye that has limited fluorescence signal in aqueous solution but increases fluorescence intensity upon binding phospholipids exposed at a membrane lesion. Dystrophic myofibers incubated with SBI-3h antibody showed decreased FM 4–64 dye uptake 260 s after laser-induced membrane injury compared to PBS and IgG controls, consistent with protection from microinjury in vitro (Fig. 4).

To determine the effect of anti-LTBP4 antibody administration on sarcolemmal injury in vivo, *mdx* mice were pretreated for 2 weeks with anti-LTBP4 antibody (10 mg/kg) or PBS. After 2 weeks of antibody or vehicle intraperitoneal injection, mice were injected with Evan’s blue dye, and then the TA muscle was injured by toxin injection (fig. S3). The muscle was allowed to recover for 3 hours after injury to evaluate the immediate effects after injury. Evan’s blue dye was used as a measure of injury, as dye is excluded from muscle with an intact sarcolemma. Anti-LTBP4–treated muscle had a reduction in percentage of injury area and decreased dye fluorescence compared to controls (fig. S3).

### Anti-LTBP4 enhances recovery from muscle injury

**Fig. 5.**
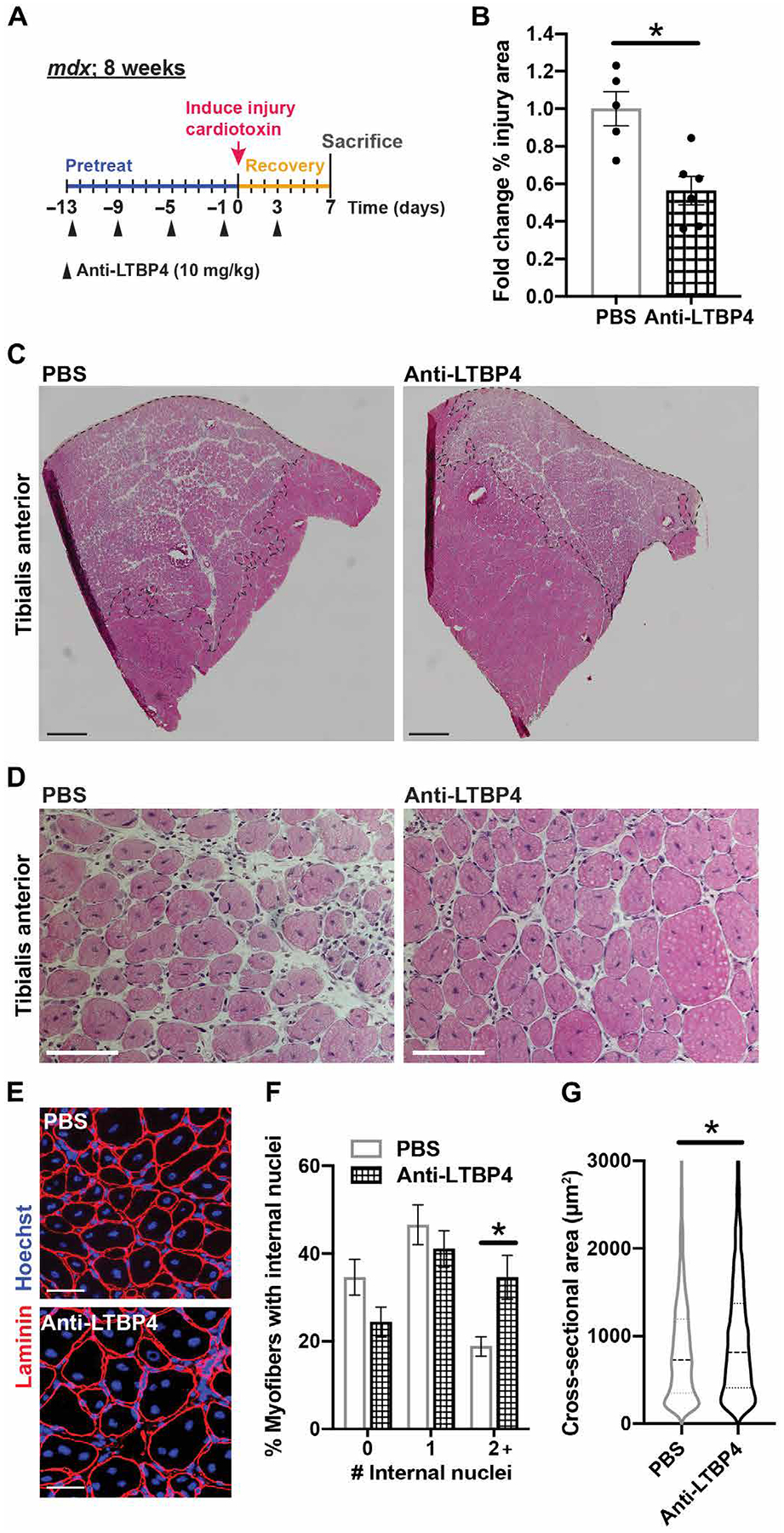
Anti-LTBP4 enhances recovery from muscle injury. (**A**) *mdx* mice were pretreated with anti-LTBP4 antibody (10 mg/kg) or PBS every 4 days for 2 weeks and once after cardiotoxin-induced muscle injury on day 3. The injured TA muscle was allowed to recover for 7 days. (**B** and **C**) H and E staining of the TA muscle cross sections revealed a reduction in the area of injury (outlined with black dotted line) with anti-LTBP4 treatment compared to controls. Scale bars, 2 mm. (**D**) Representative H and E-stained images of the region of injury. Scale bars, 100 μm. (**E**) Representative immunofluorescence images of anti-laminin (red) membrane stain in the region of injury. Hoechst (blue) marks nuclei. Scale bars, 50 μm. The effect of anti-LTBP4 administration on (**F**) myofibers with two or more (2+) internal nuclei or (**G**) myofiber cross-sectional area. *P < 0.05 by *t* test (B and F) and two-way ANOVA (E). *n* ≥ 5 animals per treatment.

We evaluated the effect of anti-LTBP4 antibody treatment at a later time point after injury since later time points also reflect additional reparative processes. As above, *mdx* mice were pretreated with anti-LTBP4 antibody (10 mg/kg) or PBS for 2 weeks, and then the TA muscle was injured with toxin injection (Fig. 5A). The muscle was allowed to recover for 7 days after injury when it was then taken for analysis. Anti-LTBP4–treated muscle had a reduction in injury area compared to control (Fig. 5, B and C). Evaluation of myofibers within the injured area revealed an increased percentage of myofibers with two or more internal myonuclei in muscles treated with anti-LTBP4 compared to PBS controls (Fig. 5, D to F). In addition, muscle treated with anti-LTBP4 demonstrated an increased cross-sectional area of myofibers in the injured area compared to controls (Fig. 5, D, E and G). Together, these data show that anti-LTBP4 antibody enhances muscle recovery after injury in vivo.

### Long-term anti-LTBP4 dosing increases myofiber size and enhances muscle function

**Fig. 6.**
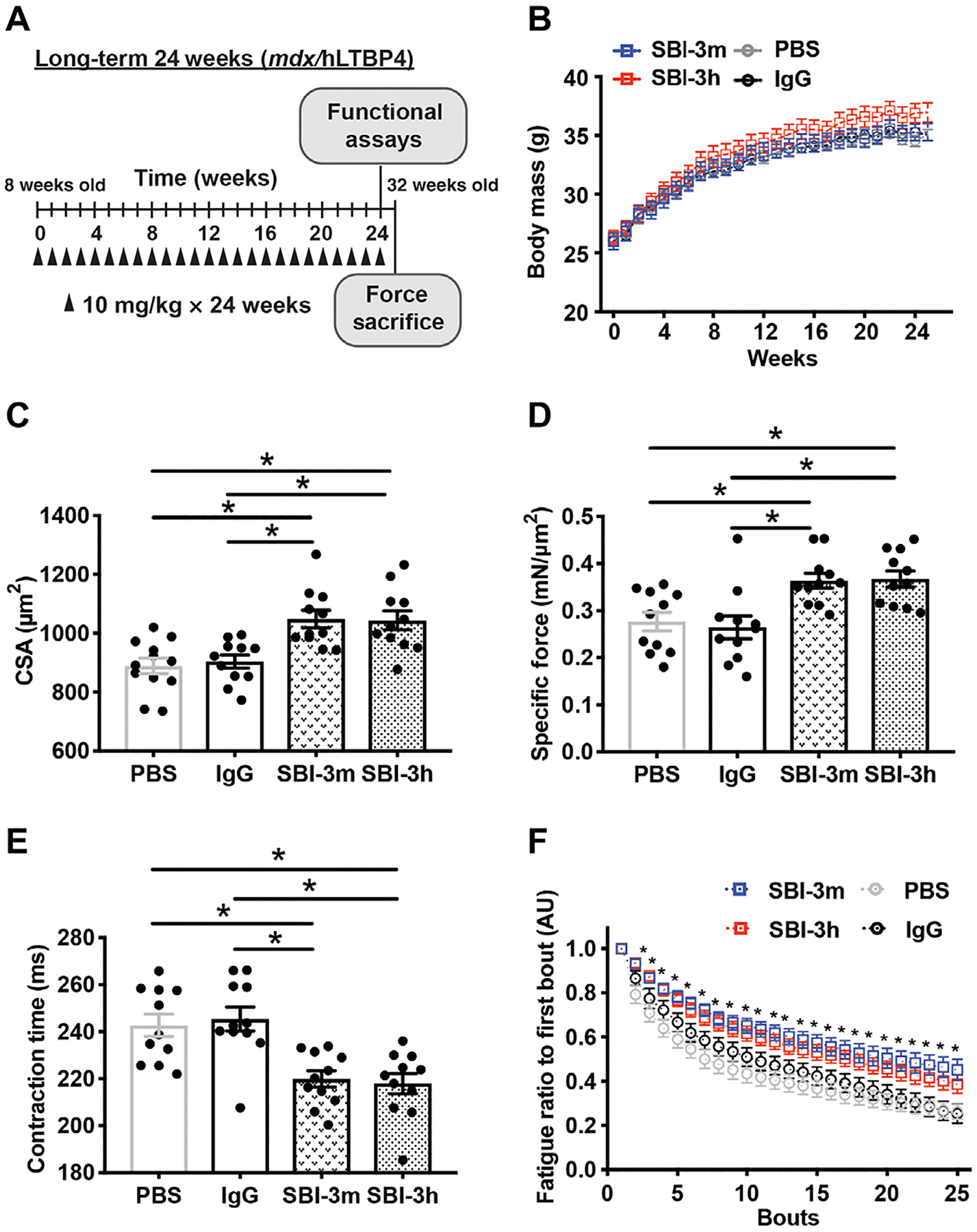
Long-term treatment with the anti-LTBP4 antibody increased muscle size and enhanced muscle performance in dystrophic mice. (**A**) *mdx*/hLTP4 male mice were injected intraperitoneally with either PBS, human IgG control, SBI-3h, or mouse anti-LTBP4 antibody (SBI-3m) at 10 mg/kg once per week for 24 weeks starting at 8 weeks of age and completing at 32 weeks of age. (**B**) Whole body weight after anti-LTBP4 treatment compared to PBS or IgG control. (**C**) Myofiber cross-sectional area (CSA) in mice injected with SBI-3m or SBI-3h compared to PBS and control IgG-injected animals. (**D**) Specific force was increased in anti-LTBP4 antibody–treated mice compared to PBS and IgG controls. (**E**) Contraction time was faster in anti-LTBP4 antibody–treated muscle compared to PBS and IgG controls. (**F**) Muscles from anti-LTBP4 antibody injected mice displayed improved resistance to fatigue compared to PBS and IgG controls. *P < 0.05 by one- or two-way ANOVA. *n* ≥ 11 mice per group.

Muscular dystrophy is progressive over time. We performed a long-term study evaluating animals beginning at 8 weeks of age and continuing to 32 weeks of age to assess pathological features, such as fibrosis, which accumulate over months. *mdx*/hLTBP4 mice were injected into the intraperitoneal cavity with SBI-3h or SBI-3m, engineered to harbor the mouse Fc region (fig. S1), at a dosage of 10 mg/kg, once weekly, for a total of 24 consecutive weeks (Fig. 6A). PBS and human IgG antibody were administered as controls once per week. Mice receiving 24-week administration of SBI-3h antibody trended toward increased body mass (*P* = 0.07) (Fig. 6B). Forelimb grip strength was not different between groups (table S1). However, TA myofiber cross-sectional area was larger after long-term SBI-3h or SBI-3m administration compared to PBS and IgG controls (Fig. 6C).

In situ tetanic force analysis of TA muscles at the 24-week end point showed increased muscle strength in SBI-3h– and SBI-3m–treated animals. Specific force was increased in both mouse and human anti-LTBP4 antibody–treated mice compared to PBS and IgG controls (Fig. 6D). In addition, contraction time was faster after SBI-3h or SBI-3m compared to PBS and IgG controls (Fig. 6E). Fatigue curves over 25 consecutive isometric contractions showed greater resistance to fatigue after SBI-3h or SBI-3m injections (Fig. 6F). Thus, long-term, once-weekly anti-LTBP4 antibody dosing improved mass, tetanic force, contraction time, and fatigue resistance in dystrophic muscle, as compared to control treatments.

Cardiomyopathy is a pathological feature of DMD in mice and humans (21) but typically does not occur in *mdx* mice until much later in age (22). Left ventricular fractional shortening (FS %) was increased in mice treated with SBI-3m compared to PBS controls (fig. S4A). However, it must be noted that FS was normal in untreated *mdx*/hLTBP4 at this age, with no difference between mdx/hLTBP4 hearts and background-matched wild-type mice injected with PBS control. In addition, PR and QRS intervals were not different between treatment groups or controls (fig. S4, B and C). We detected SBI-3h in other tissues such as heart, lung, and kidney, consistent with the known broad expression of Ltbp4. We did not observe any unusual toxicity or untoward effects in treated mice or control-injected animals. Together, these data indicate that long-term treatment of *mdx*/hLTBP4 muscle with anti-LTBP4 antibody increases muscle mass and force production without evident cardiotoxicity.

### Long-term anti-LTBP4 dosing reduces muscle fibrosis and improves respiratory function

**Fig. 7.**
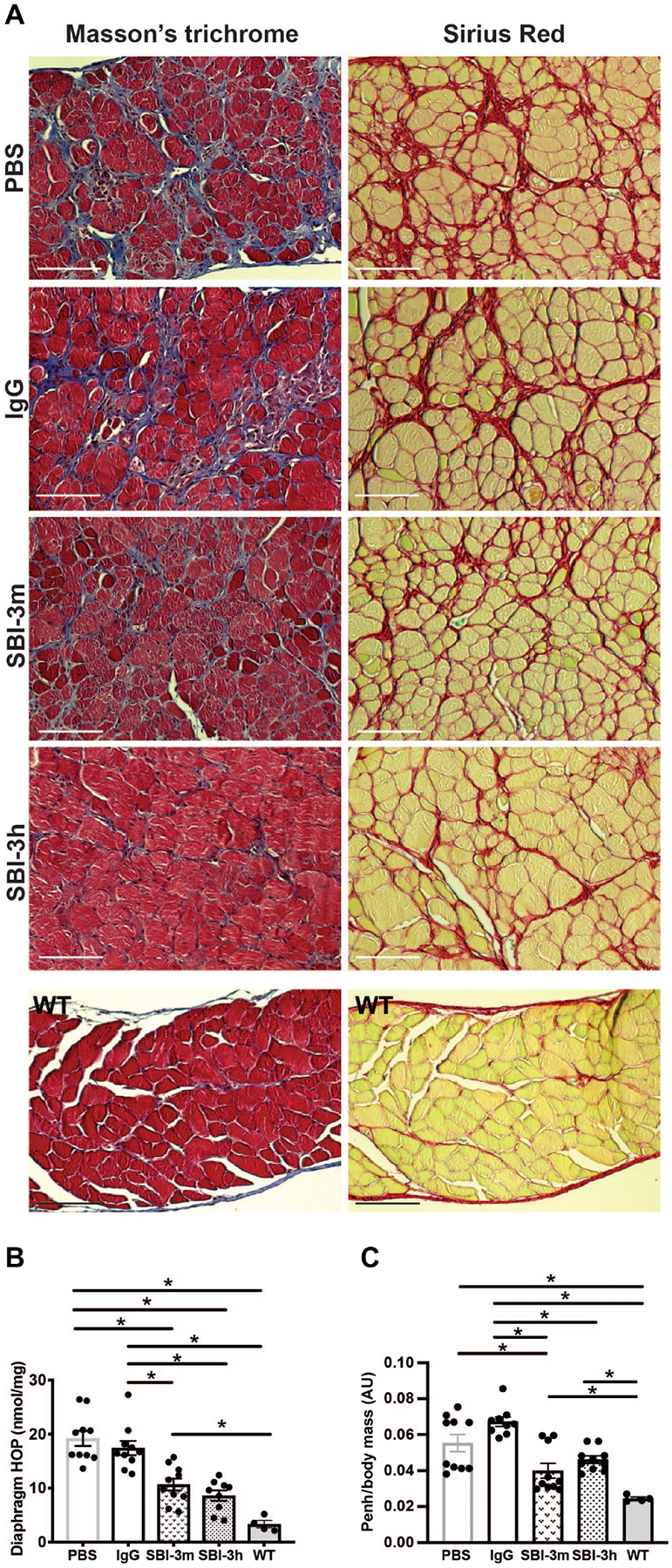
Long-term treatment with anti-LTBP4 antibody dosing reduced muscle fibrosis in dystrophic mice. (**A**) Fibrosis as shown by Masson’s trichrome (blue) or collagen content as seen by Sirius Red (red) after mouse anti-LTBP4 (SBI-3m) or human anti-LTBP4 (SBI-3h) antibody treatment compared to PBS control or human IgG controls in mdx/hLTBP4 diaphragm muscle. Normal wild-type diaphragm staining is included for comparison. Scale bars, 100 μm. (**B**) Hydroxyproline (HOP) concentration after mouse anti-LTBP4 (SBI-3m) or human anti-LTBP4 (SBI-3h) antibody treatment compared to PBS and IgG human controls. Hydroxyproline concentration after SBI-3h antibody treatment was reduced so that it was not statistically different from wild type. (**C**) Whole-body plethysmography measures respiratory muscle function. Enhanced pause (Penh)/body mass was reduced (improved) with SBI-3m and SBI-3h treatment compared to controls. *P < 0.05 by one-way ANOVA. *n* ≥ 9 mice per group, except *n* = 4 WT controls.

TGFβ activation is a potent stimulator of fibrosis in multiple tissues including muscle. To determine the effect of anti-LTBP4 antibodies on fibrosis, the diaphragm was evaluated by Masson’s trichrome and Sirius Red staining. SBI-3m and SBI-3h treatment decreased fibrosis/collagen content in diaphragm muscles compared to PBS and IgG controls (Fig. 7A). To further quantify the reduction in fibrosis, multiple muscle groups from 24-week treated mice were evaluated for hydroxyproline content, a measure of collagen deposition and fibrotic scar formation (7, 23, 24). Long-term treatment with SBI-3h or SBI-3m reduced hydroxyproline content compared to PBS and IgG controls in diaphragm (Fig. 7B), gastrocnemius/soleus, and abdominal tissues (fig. S5, A and B). Mouse and human anti-LTBP4 antibody administration reduced hydroxyproline concentration in several muscle groups so that they were not different from wild-type PBS control (Fig. 7B and fig. S5, A and B). As a measure of respiratory muscle function, whole-body plethysmography was performed, and enhanced pause (Penh) was calculated as (Penh = (PEF/PIF) * Pause). Enhanced pause (Penh)/body mass was decreased (improved) in mice treated with SBI-3m or SBI-3h for 24 weeks compared to controls. Both Evans’ blue dye uptake and serum creatine kinase (CK) were highly variable within each treatment group with no differences noted between groups with the exception of wild-type control mice (fig. S5, C and D).

Recognizing that long-term dosing of mice with a human antibody may activate an immune response that could affect the mouse dystrophic phenotype, we evaluated immune cell activation through quantitation of expression of the cytotoxic immune cell marker granzyme B (*Gzmb*) (25). GZMB is not highly expressed in muscle tissue [0.28 transcripts per million (TPM) compared to spleen (22 TPM) and blood (78 TPM) (GTEx Portal accessed on 20 March 2019)]. After long-term administration, spleen *Gzmb* mRNA did not differ between the PBS and SBI-3m groups, while *Gzmb* was up-regulated in spleens of mice treated with human IgG and SBI-3h (fig. S6), indicating at least partial immune cell activation in response to human IgG administration. This was not observed in skeletal muscle tissue, although there was a reduction in *Gzmb* mRNA observed in the SBI-3m–treated animals as compared to PBS and SBI-3h (fig. S6). Thus, the immune system may have been differentially activated in the human IgG-exposed mice, but this activation did not appear to inhibit the anti-LTBP4 antibody on muscle.

To verify that SBI-3h was present in long-term treated muscle, TA muscle from injected mice was sectioned and stained with anti-human IgG secondary antibody. Mice had received their last injection 7 days before harvest. Cross-sectional and longitudinal imaging confirmed the presence of human antibody in SBI-3h–treated muscles in a costameric pattern, while no human IgG signal was detected in SBI-3m, PBS, or control human IgG-injected muscles (fig. S7). Together, these data indicate that treatment with anti-LTBP4 antibody is effective in increasing muscle performance and decreasing fibrosis in a preclinical mouse model of DMD.

### Antibody dosing in combination with prednisone further enhances muscle function and protects against injury in *mdx* mice

**Fig. 8.**
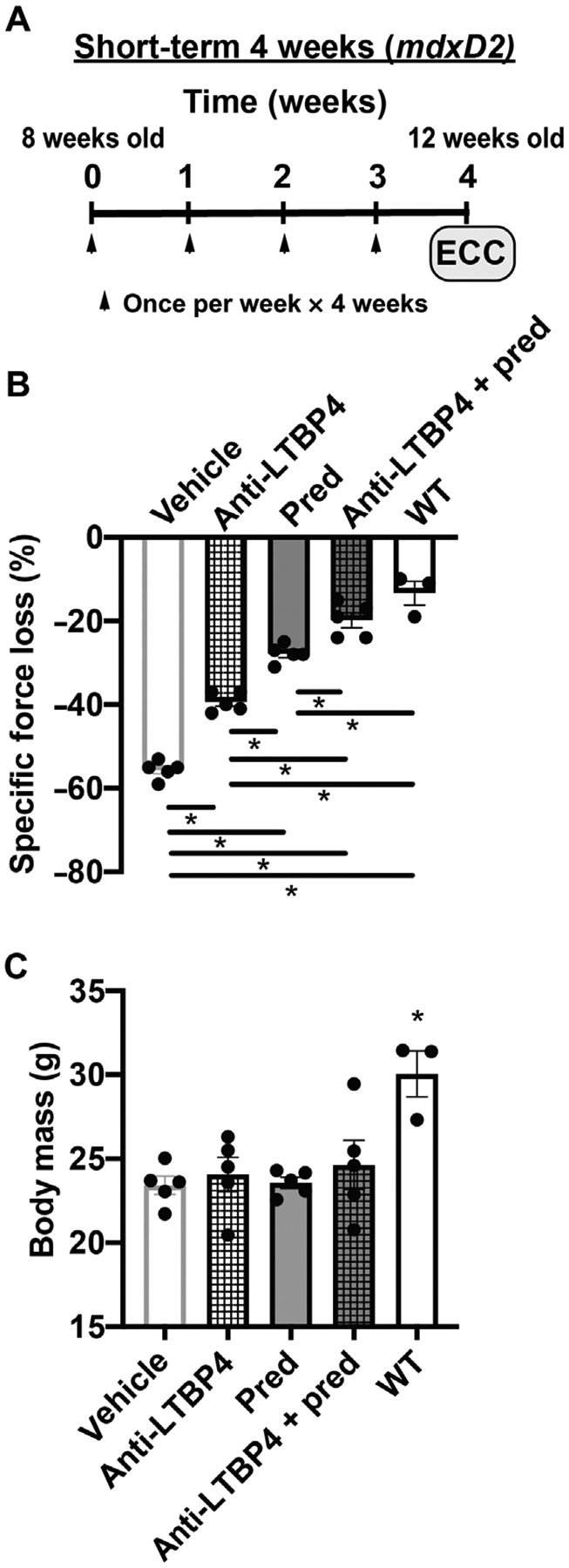
Short-term in vivo treatment with anti-LTBP4 human antibody in combination with prednisone enhanced muscle performance and protected against injury in dystrophic mice. (**A**) *mdxD2* male mice were injected intraperitoneally with either dimethyl sulfoxide (DMSO) vehicle, anti-LTBP4 at 20 mg/kg, prednisone at 1 mg/kg, or antibody (20 mg/kg) and prednisone (1 mg/kg). Injections were weekly for 4 weeks starting at 8 weeks of age and completing at 12 weeks of age. Arrowheads indicate injection timing. (**B**) Treatment with anti-LTBP4 antibody co-administered with prednisone attenuated force loss in treated TA muscles to be similar to WT controls. (**C**) Body mass was not altered after 4 weeks of treatment. *P < 0.05 by one-way ANOVA. *n* = 5 mice per *mdxD2* group. *n* = 3 WT controls.

A drug-genotype interaction was previously observed in steroid-treated patients with DMD. Specifically, glucocorticoid-treated patients with DMD who also had the protective *LTBP4* allele showed the longest ambulatory duration compared to other LTBP4 alleles with and without steroids (10). These findings support a synergistic effect between prednisone and LTBP4. To better assess this relationship, we conducted a 4-week study of *mdxD2* mice with and without prednisone. *mdxD2* mice are a more severe model of DMD as compared to the *mdx* strain on the C57 background (26). Mice were injected once per week with prednisone at 1 mg/kg with anti-LTBP4 antibody at 20 mg/kg (Fig. 8A). After 4 weeks of injection, TA muscle underwent in situ ECC injury as described above. Mice coinjected with anti-LTBP4 antibody and prednisone maintained force better after in situ ECC-induced injury compared to prednisone alone, antibody alone, and dimethyl sulfoxide controls (Fig. 8B). Body mass was not altered by any treatment (Fig. 8C). The *mdxD2* animals treated with anti-LTBP4 antibody and prednisone displayed a similar response to wild-type D2 animals, suggesting near-complete correction of this deficit with antibody and prednisone. Thus, 4 weeks of co-administration of anti-LTBP4 antibody in combination with prednisone acts synergistically to reduce the susceptibility of dystrophic muscle to ECC-induced force loss and enhance muscle performance.

## DISCUSSION

We evaluated the in vivo efficacy of a potential clinical biologic agent aimed at reducing fibrosis and promoting growth in muscular dystrophy. This preclinical testing was carried out in *mdx* mice as well as *mdx* mice expressing human LTBP4 with concordant results in short- and long-term dosing schemes. These data are consistent with the reactivity of anti-LTBP4 antibodies against both human and mouse LTBP4. LTBP4 target engagement was observed in a costameric pattern across myofibers consistent with target engagement. Detection of the human antibody on unfixed, nonpermeabilized myofibers indicates that the LTBP4 target protein is found on the external face of myofibers. This costameric pattern was observed within a day of a single injection and lasted up to 3 weeks, which was the longest time point evaluated. The antibody accumulated within the extracellular matrix with increasing numbers of injections, although further studies will be required to more precisely quantify the accumulation of antibody over time.

The reduction in fibrosis seen with anti-LTBP4 antibody exposure reflects blocking TGFβ action. Aberrant TGFβ signaling is a driver of fibrosis development after injury and in chronic disease states, such as DMD [(27), and reviewed in (28)]. Nelson and colleagues previously showed that long-term treatment with a pan-TGFβ antibody (1D11) improved respiratory function and reduced diaphragm fibrosis by 20% in *mdx* mice (29). Furthermore, halofuginone, an inhibitor of TGFβ-induced SMAD3 phosphorylation, was shown to prevent the accumulation of fibrosis in *mdx* muscle over 8 weeks of treatment (30, 31). Continuous dosing of halofuginone was required to sustain an antifibrotic effect, as fibrosis increased after discontinuation of the agent, illustrating the continuous and progressive nature of dystrophic fibrosis.

Fibrosis formation is driven not only by TGFβ signaling but also by other pathways including myostatin and nuclear factor kB (NF-κB). Myostatin is a TGFβ family member that negatively regulates muscle growth (32). Inhibiting myostatin signaling using adeno-associated viral expression of a secreted myostatin dominant-negative peptide increased muscle mass and reduced muscle fibrosis in a Golden Retriever model of muscular dystrophy (GRMD) after 13 months of exposure (33). Activation of NF-κB in muscle results in muscle loss, inflammation, fibrosis, and reduced regeneration, with NF-κB activity correlating with disease severity (34). Edasalonexent and analog CAT-1041 are potent NF-κB inhibitors. Blockade of NF-κB with these agents reduced fibrosis in *mdx* mice after 25 weeks of treatment and GRMD dogs after 9 months of treatment (35). These data underscore the duration needed to inhibit fibrosis formation. Whether any antibody or the anti-LTBP4 antibodies act differentially on muscle groups related to delivery from blood flow in muscle groups is not known. The degree of fibrosis reduction appeared comparable across the muscle groups evaluated. LTBP4 binds TGFβ1, TGFβ2, and TGFβ3, as well as myostatin (5, 6). We hypothesize that the combination of targeting TGFβs and myostatin due to enhanced LTBP4 stabilization may provide increased benefit compared to targeting single fibrotic mediators.

Enhanced membrane stability in DMD and sarcoglycan-mediated limb girdle muscular dystrophy (LGMD) would limit disease progression as membrane disruption is a primary deficit in these disorders. LTBP4’s position on the exterior of the myofiber may support a direct role in promoting a sarcolemma more resistant to breakdown (5, 9). Overexpression of LTBP4 was previously observed to promote membrane stability (5, 18). The finding that 4 weeks of in vivo administration of anti-LTBP4 antibody reduced the susceptibility of *mdx* muscle to force loss from eccentric-contraction induced injury also supports a role in membrane stabilization. The activin type IIB receptor is a TGFβ superfamily receptor. Treatment with a soluble receptor/FC fusion (sActRIIB) decoy protein did not improve force loss after ECC in *mdx* mice. In addition, myostatin blockade did not improve force loss after ECC in *mdx* mice or a model of LGMD2C (36, 37). The strategy of LTBP4 antibody stabilization to increase TGFβ sequestration is unique in that LTBP4 stabilization is hypothesized to locally reduce TGFβ signaling at the costamere and in tissues that endogenously express LTBP4. This is unlike other strategies that globally neutralize TGFβ or myostatin signaling. Targeting signaling in specific locations, such as the costamere, may differentially modulate the ECM and membrane, resulting in protection against injury.

Elevated serum CK is a diagnostic biomarker of muscle injury and breakdown. However, its use as a pharmacodynamic biomarker is more controversial due to the high intraindividual variation and activity-dependent fluctuations (38). The effects of TGFβ-neutralizing antibody treatments and antimyostatin agents on serum CK are mixed, with treatments showing a reduction in only a subset of studies (33, 36, 37, 39, 40). After treatment with anti-LTBP4 antibody, we did not observe a reduction in serum CK or dye uptake into muscle, although the high variability in these readouts and their dependence on activity should be noted. It is plausible that anti-LTBP4–treated mice increased daily activity compared to untreated controls, which might promote enhanced CK release. Further characterization of mouse activity with anti-LTBP4 treatment would be necessary to address this hypothesis. We found that anti-LTBP4 treatment alone in *mdx* mice improved contraction-induced injury, reducing force loss by 30%, compared to untreated *mdx* controls. However, anti-LTBP4–treated mice still exhibited ~3 times more injury than healthy wild-type controls. This amount of underlying muscle damage is likely sufficient to elevate creatine kinase.

We found that the combinatorial effect of a 4-week anti-LTBP4 antibody treatment in conjunction with steroids further reduced percent force loss after contraction-induced injury, as to be not different from wild type. Further evaluation of this combinatorial approach after long-term treatment, specifically measuring creatine kinase as a biomarker of muscle stability, is warranted, as steroid use is the standard of care for most Duchenne patients. Work from others has suggested that IgG on its own may have benefit (41). However, we controlled for this possibility using human IgG in addition to a PBS arm and saw improvement above these controls.

For DMD, ongoing efforts are aimed at restoring dystrophin expression, through exon skipping, gene therapy, or gene editing (42–46). It should be feasible to combine anti-LTBP4 therapy with these other approaches, as correction, even with gene therapy, is expected to be incomplete. In addition, anti-LTBP4 therapy should be useful across other forms of muscular dystrophy, especially those that share similar pathology. A protein biologic, anti-LTBP4 might be useful as an antifibrotic for other disorders in which tissue or cellular dysfunction arises from increased extracellular matrix with excess TGFβ activity.

## MATERIALS AND METHODS

### Study design

For both short- and long-term studies, mice were weighed at study start and distributed to ensure equal average weights among each treatment group at study onset. Once assigned to treatment groups, animals were then distributed for housing and injection days so that treatment groups were intermingled to avoid being segregated by treatment grouping and to help ensure blinding of treatment group to investigators handling the mice. Wild-type mice were housed in a single cage. Experimental antibodies and controls (PBS or control IgG) were prealiquoted and then handed to investigators blinded to content for all injections. All studies were carried out blinded to the assigned treatment group. Unblinding of injection content occurred only after analyses were completed. Sample sizes for in vivo were selected to obtain ~20% differences between experimental interventions and vehicle controls based on pilot dosing studies, anticipated biological variability, and potential genotype-related mortality. Reporting guidelines adhered to EQUATOR guidelines for preclinical animal studies (ARRIVE).

### Animals

Mice were housed in a specific pathogen–free facility in accordance with Northwestern’s Institutional Animal Care and Use Committee regulations and National Institutes of Health (NIH) *Guide for the Care and Use of Laboratory Animals*. *mdx* mice were obtained from the Jackson Laboratory (Bar Harbor ME). *mdx*/hLTBP4 were generated as previously described (9). Grip strength, force measurements, hydroxyproline measurements, and echocardiography were performed as previously described (47) with detailed methods in the Supplementary Materials.

### Sequence comparison and protein schematics

Human IgG antibodies were selected on the basis of reactivity to the peptide (GFLPTHRLEPRPEPRPDPRPGPELPLPSIPAWTGPEIPESG).

### Expression constructs/HEK expression

The long isoform of mouse *LTBP4-L* (uc009fvt.2, transcript variant 1) was generated to contain a C-terminal Xpress tag as previously described (5). Full-length human LTBP4 was generated as previously described (6).

### EDL myofiber isolation

EDL muscles were dissected and placed into relaxing solution [100 mM BES, 15 mM creatine phosphate, 5 mM dithiothreitol, 17 mM propionic acid, 4.74 mM adenosine triphosphate, 7 mM ethylene glycol tetraacetic acid, 5.43 mM magnesium chloride, and 0.02 mM calcium chloride, adjusted to pH 7.2 with sodium hydroxide] (9). While in relaxing solution, small fiber bundles were teased apart and isolated myofibers were rinsed in 1× PBS (14190–250; Thermo Fisher Scientific, Waltham, MA). Immunofluorescence was performed as outlined below.

### Cardiotoxin injury and analysis

*mdx*C57 mice were injected with anti-LTBP4 antibody (10 mg/kg) or PBS as described above. Mice were weighed at study start, and cohorts were created controlling for body mass (mean of 25 g). Mice were injected for 2 weeks, every 4 days, before cardiotoxin injury (days −13, −9, −5, and −1) for both the 3-hour and 7-day cohorts, and then once after injury on day 3, for those mice evaluated 7 days after injection. Cardiotoxin injury was performed as previously described (48, 49). One lot of cardiotoxin was reconstituted and stored at −20°C for use across all experiments. On the day of use, the stock solution was thawed and diluted with PBS to a concentration of 10 μM. Twenty microliters of 10 μM cardiotoxin solution was injected into the TA muscle of sedated animals (3% isoflurane, 0.8 liter/min O_2_) using an insulin syringe. Cardiotoxin was released down the midline of the muscle to induce a homogeneous area of injury at the center of the muscle. Mice were allowed to recover for 3 hours or 7 days after cardiotoxin injection, and then muscle was harvested and flash-frozen in liquid nitrogen. For the cohort evaluated at 3 hours after injection, mice were injected retro-orbitally with 10 μM Evan’s blue dye (5 μl/g; E2129; Sigma-Aldrich, St. Louis, MO), 2 hours before cardiotoxin injection.

### FDB myofiber isolation and laser injury

Fibers were dissected and laser-damaged as previously described (19, 20). Briefly, fibers were dissociated in 0.2% bovine serum albumin and collagenase type II (17101, Thermo Fisher Scientific, Waltham, MA) for 60 min at 37°C in 10% CO_2_. Fibers were then moved to Ringers solution and placed on MatTek confocal microscopy dishes (P35G-1.5–14-C, MatTek, Ashland MA). Myofibers were incubated with or without anti-LTBP4 antibody for 3 hours. FM 4–64 dye (T-13320, Molecular Probes, Grand Island, NY) was added to a final concentration of 2.5 μM before imaging. Fibers were irradiated at room temperature using a Nikon A1R laser scanning confocal equipped with GaSP detectors through a 60× Apo lambda 1.4 numerical aperture objective driven by Nikon Elements AR software. We ablated a single pixel set as 120 nm (0.0144 μm^2^) using the 405-nm laser at 100% power for up to 5 s. Images were acquired as follows: One image was acquired before damage (preinjury), 1 image upon laser damage (0 s), 10 images every 2 s after damage, and then 1 image every 10 s for up to 4 min after injury. Z-stack projections were acquired from approximately 35 consecutive acquisitions with 125-nm intervals between each step, using the z-stack rendering built-in tool in NIS-elements AR (Nikon). Relative endpoint fluorescence at the lesion was assessed using FIJI (NIH).

### Protease cleavage assay

Recombinant human LTBP4 was incubated with anti-LTBP4 antibody for 1 hour at 4°C and then exposed to varying concentrations of elastase (U) (E7885; Sigma-Aldrich, St. Louis, MO) for 15 min at 37°C. The digestion products were separated by 4 to 15% precast polyacrylamide gel electrophoresis (4561086, Bio-Rad) and transferred to polyvinylidene fluoride membrane (1620177, Bio-Rad). Membrane was blocked in StartingBlock T20 (tris-buffered saline) Blocking Buffer for 1 hour at room temperature and then incubated overnight in His Tag primary antibody used 1:1000 (MAB050; R&D Systems, Minneapolis, MN). Secondary antibody was used at 1:2500 (115-035-003; Jackson ImmunoResearch, West Grove, PA). Pierce Pico and Femto chemiluminescent substrate was applied to membranes, and membranes were visualized using an Invitrogen iBright CL1000 Imaging System. Immunoblot bands were quantified using FIJI gel analysis tools (NIH).

### Statistical analysis

Statistical analyses were performed using Prism software v7.0a (GraphPad, La Jolla, CA). When comparing two groups, two-tailed Student’s *t* test with Welch’s correction (unequal variances) was used. When comparing three or more groups of data for only one variable, one-way ANOVA with Tukey multicomparison was used. When comparing data groups for more than one related variable, two-way ANOVA was performed. *P* value less than or equal to 0.05 was considered significant. Data were presented as single values where appropriate. In analyses pooling larger data point sets per group, Tukey distribution bars were used to emphasize data range distribution. Analyses pooling data points over time were presented as marked line plots. Error bars represent ±SEM.

## Supplementary Material

Supplementary.pdf

## Data Availability

All data associated with this study are present in the paper or the Supplementary Materials. Requests for anti-LTBP4 antibodies may be considered for noncommercial use based on availability of supply and through a material transfer agreement by contacting E.M.M.
