# Supplementary material for: Anti-latent TGFβ binding protein 4 antibody improves muscle function and reduces muscle fibrosis in muscular dystrophy
Source: Sci Transl Med. Author manuscript; Available in PMC 2022 Oct 13. (PMC9559620; doi:10.1126/scitranslmed.abf0376)
Supplement: Supplementary.pdf [file NIHMS1841393-supplement-Supplementary_pdf.pdf]

Science Translational Medicine
AAAS

Supplementary Materials for

Anti-latent TGFβ binding protein 4 antibody improves muscle function and reduces muscle fibrosis in muscular dystrophy

Alexis R. Demonbreun *et al.*

Corresponding author: Alexis R. Demonbreun, alexis.demonbreun@northwestern.edu;
Elizabeth M. McNally, elizabeth.mcnally@northwestern.edu

Sci. Transl. Med. **13**, eabf0376 (2021)
DOI: 10.1126/scitranslmed.abf0376

The PDF file includes:

Materials and Methods
Figs. S1 to S7
Table S1
Legend for data file S1
Reference (*50*)

Other Supplementary Material for this manuscript includes the following:

Data file S1

Supplementary Materials for DEMONBREUN ET AL.

Materials and Methods

Animals. Mice were fed *ad libitum* and maintained on a 14/10-hour light/dark cycle. Male *mdx*, *mdx*/hLTBP4, or wildtype B6 mice were used for reported experiments. Male *mdx* mice were 8 weeks of age at the beginning of the short-term (4 week) studies including laser injury and eccentric contraction. *mdx*/hLTBP4 mice we bred with *mdx* females through 2-3 generations to expand the colony and produce sufficient number of mice of the appropriate age to carry out concurrent testing of four different treatment groups for the long-term study. Male *mdx*/hLTBP4 mice and wildtype B6 control mice were 8 weeks of age at the initiation of the 24-week study. Male *mdxD2* were obtained from Jackson Laboratories, wildtype D2 mice were used as controls. Mice were 8 weeks of age at the beginning of the short-term (4 week) prednisone/antibody study.

Antibody and prednisone treatments. For all studies, mice were injected once a week via intraperitoneal injection. The total volume injected did not exceed 225µl or 350µl per individual injection for short- and long-term studies, respectively. For antibody time course studies, mice were injected IP with 10mg/kg human anti-LTBP4 once and sacrificed 1, 7, 14, or 21 days later. Control mice were injected with PBS once and sacrificed 1 day later. Individualized aliquots were prepared by a technician in a sterile laboratory hood and kept covered on ice for injection by a different technician who was blinded to content. Sterile BD™ Micro-Fine IV Insulin Syringes (Cat #14-829-1A; Thermo Fisher Scientific) were used to inject the intraperitoneal cavity of non-sedated animals. Injections were performed blinded to treatment group. For prednisone injections, prednisone (p6254, Sigma-Aldrich) was resuspended in DMSO (D2650, Sigma-Aldrich) at 5 mg/ml. DMSO alone was used as a vehicle control. Dosing was based on weekly body weights (1 mg/kg body weight). For co-administration with anti-LTBP4 antibody, reconstituted prednisone was added to the antibody solution. Injection volume was normalized with PBS as described above. Mice were injected via intraperitoneal injection between 7 and 8 am. Wildtype D2 mice were injected with DMSO as a comparator group.

Expression constructs/HEK expression. Briefly LTBP4 (NM_001042544.1) cDNA clone was purchased from Origene (catalog number SC311430). The Xpress epitope tag (DLYDDDDK) was added onto the 5'/N-terminus of human LTBP4. HEK293-T cells were obtained from ATCC (CRL-11268). Transfections were performed using FuGENE HD Transfection Reagent (E2311; Promega, Madison, WI) at a DNA:FuGENE ratio of 1:3. Opti-MEM was replaced with serum-rich media 24 hours after transfection and cells were harvested 3 days after transfection.

Immunoblotting. Muscles were lysed in whole tissue lysis buffer (50mM HEPES pH 7.5, 150mM NaCl, 2mM EDTA, 10mM NaF, 10mM Na-pyrophosphate, 10% glycerol, 1% Triton X-100, 1 mM phenyl-methylsulfonyl fluoride (PMSF), 1X Roche cOmplete™ Protease Inhibitor Cocktail (11697498001 CO-RO; Roche). HEK cells were lysed in cell lysis buffer (150 mM NaCl, 50 mM Tris HCl (pH 8), 25 mM β-glycerophosphate, 10 mM sodium pyrophosphate, 2 mM EDTA, 0.1% Triton X-100, 1x cOmplete Protease Inhibitor tablet, and 1 mM PMSF). Protein concentration was determined using Quick Start™ Bradford protein assay (5000205 Bio-Rad Laboratories). Recombinant human LTBP4 (rhLTBP4) was purchased from R&D Systems (8068-LT-050). Proteins were separated on a 4–15% Mini-PROTEAN® TGX™ Precast Protein Gels, 15-well, 15 µl (4561086; Bio-Rad Laboratories) and transferred to Immun-Blot PVDF membranes for protein blotting (1620177; Bio-Rad Laboratories). Blocking and antibody incubations were done using StartingBlock T20 (TBS) Blocking Buffer (37543; Thermo Fisher Scientific). Anti-Xpress antibodies were generated as described (*6*). Primary antibodies were

used are as follows: monoclonal anti-LTBP4 at 1:500; rabbit polyclonal anti-Xpress at 1:500. Donkey anti-human and goat anti-rabbit secondary antibodies conjugated to horseradish peroxidase (709-035-149 and 111-035-003; Jackson ImmunoResearch) were used at a dilution of 1:2500. SuperSignal™ West Pico Chemiluminescent Substrate and SuperSignal™ West Femto Maximum Sensitivity Substrate (34080 and 34096; Thermo Fisher Scientific) were applied to membranes and membranes were visualized using an Invitrogen™ iBright™ CL1000 Imaging System (A32749; Thermo Fisher Scientific). Pierce™ Reversible Protein Stain Kit for PVDF Membranes (24585; Thermo Fisher Scientific) was used to stain the blot to ensure complete transfer and equal loading. Immunoblot bands were quantified using FIJI gel analysis tools.

Immunofluorescence microscopy. For LTBP4 localization studies, myofibers were incubated in primary antibody LTBP4 (1:100) diluted in PBS plus 10% fetal bovine serum overnight at 4°C on a nutator. Control myofibers were incubated in PBS plus 10% fetal bovine serum without primary antibody. Goat anti-human IgG (H+L) Cross-Adsorbed Secondary Antibody, Alexa Fluor® 488 used at 1:2500 (A-11013; Thermo Fisher Scientific) and Hoechst 33342 used at 1:10,000 (H3570; Thermo Fisher Scientific) were incubated for 1 hour at room temperature. For detection of human LTBP4 antibody, 10µm thick muscle sections from long-term injected mice were fixed with 4% PFA made from 16% paraformaldehyde solution (15710; Electron Microscopy Sciences, Hatfield, PA). Additionally, FDB myofiber bundles were isolated and fixed in 4% cold paraformaldehyde (PFA) directly after isolation. After fixation, muscle was rinsed with PBS, blocked in PBS plus 10% fetal bovine serum for 1hr at 4C, then incubated with anti-human secondary at (1:2500) and Hoechst (1:10,000) for 1hr at room temperature. All samples were fixed in ProLong® Gold Antifade Mountant (P36930; Thermo Fisher Scientific) and imaged on a Zeiss Observer with Zen Pro software.

pSMAD1/3/5, caveolin and laminin. Ten µm thick sections from tibialis anterior muscle was fixed in 4% cold paraformaldehyde (PFA) containing PhosSTOP Phosphatase Inhibitor tablets (Cat #PHOSS-RO; Roche, Basel, Switzerland) for 10 minutes. Sections were rinsed in ice-cold PBS containing PhosSTOP, then blocked in PBS plus 10% fetal bovine serum plus 0.1% Triton X-100 for 2 hrs at 4°C. Then sections were incubated with 1:100 anti-SMAD1/3/5 (phospho S423 + S425) antibody (ab51451; Abcam) and 1:300 anti-Laminin-2 (α-2 Chain) antibody, rat monoclonal (L0663; Sigma-Aldrich) or anti-caveolin 3 antibody, mouse-monoclonal (610420, BD Biosciences) overnight at 4°C. Goat anti-rabbit IgG (H+L) Superclonal™ Secondary Antibody, Alexa Fluor® 488 at 1:2500 (A-27034; Thermo Fisher Scientific), Donkey anti-Rat IgG (H+L) Highly Crossed-Adsorbed Secondary Antibody, Alexa Fluor® 594 at 1:2500 (A-21209; Thermo Fisher Scientific, Waltham, MA), or Goat anti-Mouse IgM (Heavy chain) Cross-Adsorbed Secondary Antibody, or Alexa Fluor 594 (ThermoFisher; A-21044) and Hoechst (1:10,000) were incubated for 1 hour at room temperature. Slides were mounted in ProLong® Gold Antifade Mountant. Images were acquired on a Zeiss Observer with a 20x objective using Zen Pro software. Internal nuclei pSMAD fluorescence intensity was measured for individual internal nuclei using FIJI (NIH).

Laminin and Hoechst. Six µm thick sections from mouse tibialis anterior (TA) muscle was fixed in formalin for 10 minutes. Sections were rinsed in ice-cold PBS, then blocked in PBS plus 10% fetal bovine serum plus 1% bovine serum albumin for 1 hr at 4°C. Then sections were incubated with 1:100 anti-Laminin-2 (α-2 Chain) antibody, rat monoclonal (L0663; Sigma-Aldrich). Donkey anti-Rat IgG (H+L) Highly Crossed-Adsorbed Secondary Antibody, Alexa Fluor® 594 at 1:2500 (A-21209; Thermo Fisher Scientific) and Hoechst (1:10,000) were incubated for 1 hour at room temperature. Slides were mounted in ProLong® Gold Antifade Mountant. Images were acquired on a Keyonce microscope with a 20x objective.

Injury Analyses

Injury area 3hrs after injection. Mouse TA muscle was cross-sectioned. Four sections per muscle were saved every 30µm, spanning 120µm of muscle. Sections were fixed for 5 mins in 4% paraformaldehyde, were rinsed in PBS, and then stained with WGA-488 used at 5µg/ml (W11261;Thermo Fisher Scientific) and Hoechst 33342 used at 1:10,000 (H3570; Thermo Fisher Scientific). Slides were mounted in ProLong® Gold Antifade Mountant. The entire muscle section was imaged using the stitching feature on a Keyonce microscope using a 4x objective. All sections were imaged identically using the same exposures. Injury area (calculated as (EBD-positive area / by total muscle area) *100)) and Evan's blue dye uptake fluorescence was measured as integrated density was measured using FIJI (NIH). Surface plot profiles were generated in FIJI (NIH). Sixteen of 20 samples passed quality control and were included in the analysis. Excluded samples were as follows: one sample was excluded from analysis due to an incomplete cardiotoxin injection, two samples were excluded due to containing only a partial muscle section, and one sample was excluded that required additional processing / handling steps and subsequently was >2 standard deviations above the mean outside the 95% CI.

Analyses done on muscle seven days after injury. Mouse TA muscle was sectioned in cross-section and stained with hematoxylin and eosin (H & E). The entire muscle section was then imaged with a Keyonce microscope at 10x with the tiling feature. The region of injury was measured using FIJI (NIH) and expressed as a percentage of the total cross-sectional area of the muscle measured with FIJI(*50*). Internal nuclei were counted in the region of cardiotoxin-induced injury from laminin/Hoechst immunostained sections. Internal nuclei were counted per myofiber using FIJI (NIH). Data was expressed as % of myofibers containing x number of nuclei with >200 myofibers per mouse counted and >1000 myofibers analyzed per treatment group. Myofiber cross-sectional area was measured in the region of cardiotoxin-induced injury from laminin immunostained sections using SMASH. >350 myofibers per mouse were measured and >2500 myofibers analyzed per treatment group.

Physiologic analyses. Mice were weighed once per week over the course of 4 weeks (short-term experiments) or 24 weeks (long-term experiments). Weekly body weight informed the injected dosage volume. For long-term treated mice, grip strength, serum collection, echocardiography, and ECG were performed at study start and then once every 8 weeks for 24 weeks. Analyses were done blinded to genotype and treatment.

Body weight and muscle analysis. Mice were weighed weekly over 24 weeks. At sacrifice tibia lengths were measured, and raw body mass was normalized to the average tibia length. Muscles were removed, immediately weighed and normalized to tibia length. The midportion of the quadriceps, triceps, gastrocnemius/soleus, and gluteus/hamstrings muscle was used for histological analysis, while the remaining portion was minced and used for EBD and HOP quantitation. Excised muscles were immediately frozen in liquid nitrogen, placed in pre-cooled Nalgene cryovials and stored at -80°C or placed in Fisher HealthCare™ PROTOCOL™10% buffered formalin (23-305510; Thermo Fisher Scientific).

Grip strength. Grip strength assessments were performed at day 0 and then once every 8 weeks using a Chantillon Ametek Force Transducer in a Columbus Instruments apparatus as described (*47*). All assessments were performed by the same operator performed who was blinded to the animal's genotype.

Serum collection. Blood was collected by means of retro-orbital puncture with heparinized capillary tubes (20-362-566; Thermo Fisher Scientific) into Microtainer™ Gold Top Serum Separator (365967 Becton Dickinson) and centrifuged at 8,000 x g for 10 minutes. The plasma fractions were frozen and stored at −80°C.

Serum biomarkers. Serum creatine kinase (CK) was analyzed in triplicate for each mouse using the EnzyChrom Creatine Kinase Assay Kit (ECPK-100; BioAssay Systems) following manufacturer's instructions. Results were acquired with the Synergy HTX multi-mode plate reader (BioTek®).

Echocardiography. Echocardiography was conducted under anesthesia (0.8 L/min, 1% vaporized isoflurane in 100% O_2 using a Visual Sonics Vevo 2100 imaging system with a MS550D 22-55 MHz solid-state transducer (FujiFilm) maintaining heart rate >450 as described (*47*).

Electrocardiogram. Surface electrocardiogram recordings and analysis were performed on conscious mice using the ECGenie apparatus (Mouse Specifics). Mice were allowed to acclimatize to the ECGenie platform for five minutes and then data was collected using LabChart (ADInstruments). Four segments of 2–4 second long runs of clean ECG signal were analyzed using the ECGenie software and averaged into single data points per animal.

In-situ force and fatigue. Muscle mechanics were performed as described previously (*47*, *51*) on the tibialis anterior muscle using a Whole Mouse Test System (Cat #1300A; Aurora Scientific) with a 1N dual-action lever arm force transducer (300C-LR, Aurora Scientific). During study, animals were anesthetized (0.8 l/min of 1.5% isoflurane in 100% O_2). Both muscles per animal were assayed. Tetanic isometric contraction was induced as follows: initial delay, 0.1 sec; frequency, 200Hz; pulse width, 0.5 msec; duration, 0.5 sec; using 100mA stimulation. Length was adjusted to a fixed baseline of 50mN resting tension. Specific force equaled tetanic force normalized to muscle cross-sectional area for each muscle. Fatigue analysis used repeated tetanic contractions every 10 seconds for 25 cycles (complete exhaustion).

Eccentric contraction (ECC) induced injury. Eccentric contraction-based muscle injury was performed on *tibialis anterior* muscles of anesthetized mice (0.8 l/min of 1.5% isoflurane in 100% O_2) using a Whole Mouse Test System (1300A; Aurora Scientific) with a 1N dual-action lever arm force transducer (300C-LR, Aurora Scientific). Both muscles per animal were assayed. Eccentric contraction train was preceded and followed by two tetanic isometric contractions (initial delay, 0.1sec; frequency, 120Hz; pulse width, 0.5msec; duration, 0.5 sec; using 100mA stimulation), each executed 300 sec before last contraction. The eccentric contraction train consisted of four eccentric stimuli imparted on muscle every 100 sec using the following specifications: tetanus (100mA, 250hz, 0.1msec pulse width, 120ms duration); during tetanus, 20msec-long inward lever flexion (-5 in channel units) followed by 100msec-long outward lever flexion (+5 in channel units). Muscle tension baseline was adjusted to 50mN before the initial tetani, before the eccentric contraction train and again before final tetani. Force loss was calculated as mean initial recorded maximum tetanic force (derived from the two initial tetani) minus mean final recorded maximum tetanic force (derived from the two final tetani). Specific force was calculated as tetanic force normalized to muscle cross-sectional area assayed for each muscle from every animal.

Plethysmography. Respiratory function was measured by unanesthetized whole body plethysmography (WBP) using a Buxco Finepointe 4-site WBP (Data Sciences International) as described *(*47*)*.

Evans Blue dye uptake quantification. Mice were injected IP with 5µl/g of 10µM EBD (E2129; Sigma-Aldrich). Mice were sacrificed ~24hrs after injection. Multiple muscle groups were assessed including the abdominal, diaphragm, quadriceps, gastrocnemius/soleus, gluteus/hamstrings, and triceps and values normalized to tissue weight and kidney dye uptake. Each sample was assessed in duplicate. Absorbance was measured at 620 nm on a Synergy HTX multi-mode plate reader (BioTek®). Results are reported as arbitrary OD units/mg of tissue.

Hydroxyproline quantification. Skeletal muscle tissues (quadriceps, abdominal, gastrocnemius/soleus, gluteus/hamstrings, diaphragm and triceps) were assayed. Each sample was assessed in triplicate.

Muscle analysis. Mouse TA muscles from long-term treated mice were dissected and frozen in liquid nitrogen. Anti-laminin or anti-caveolin sarcolemmal fluorescence outlined individual myofibers and was used to assess the myofiber mean cross-sectional area (CSA) automatically using FIJI (NIH). n>11 mice per treatment from at least five fields per mouse. The percentage of fibers with central nuclei was calculated from the number of fibers containing internalized nuclei in each image/the total number of fibers counted per image, standardized as a percentage. At least 500 fibers per treatment were analyzed (n>5 from each genotype). Images were captured using a Zeiss Axiophot microscope.

Quantitative RT-PCR. Gene expression analysis was conducted on total RNA extracted with TRIzol (catalog 15596018, Life Technologies) from gastrocnemius and spleen tissues per manufacturer's instructions. RNA was isolated from approximately 50 mg tissue and 2µg RNA was used per reverse transcription reaction. Each cDNA reaction was obtained using qSCRIPT cDNA supermix assay in 20µl reactions per manufacturer's instructions (catalog 95048, QuantaBio). cDNA was diluted 1:10, and 2µl was used per 10 µl qPCR reaction. Each 10 µl qPCR reaction contained 100 nM primers and 5µl iTaq SYBR Green Mix (catalog 1725124, Bio-Rad). Primers to *gzmb*: GZMB Fw- ACAAAGGCAGGGGAGATCAT and GZMB Rev-CGAATAAGGAAGCCCCCACA. RN45S was used as a control (RN45S Fw: gtaacccgttgaacccgatt, RN45S Rev: ccatccaatcggtagtagcg). SybrGreen Fluorescence was quantitated using the CFX96 Real-Time System (Bio-Rad; thermal profile: 95C 3min, 40 cycles of 95C 15sec, 60C 45sec) and gene expression values were analyzed as fold change to control tissues.

Histology. Masson's Trichrome and Sirius Red Staining were performed on mdx/hLTBP4 and WT diaphragm muscle per manufacturer's protocols. Sections were then imaged at 10x on a Keyonce microscope, at least 3 representative images per mouse per stain.

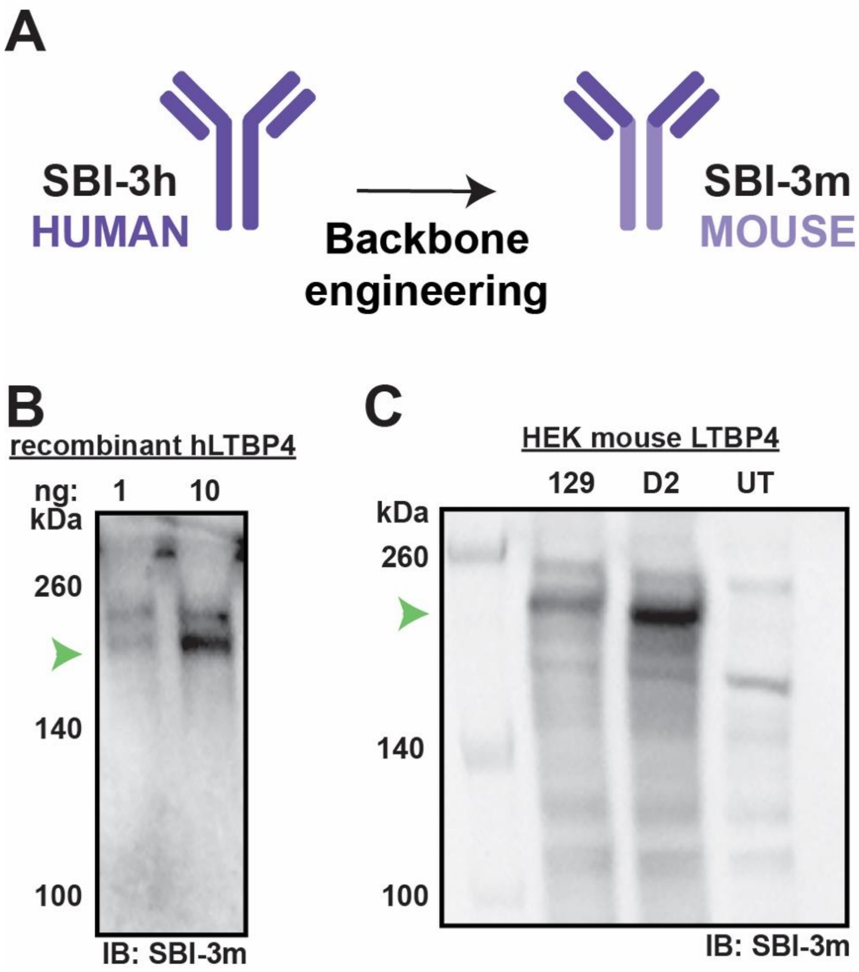

Figure S1. Mouse anti-LTBP4 monoclonal SBI-3m antibody recognizes human and mouse LTBP4. LTBP4 migrates at ~160 kDa on gel electrophoresis (green arrow). **A**) Mouse anti-LTBP4 mononclonal antibody generation through backbone engineering. **B**) Mouse anti-LTBP4 antibody SBI-3m recognized recombinant human LTBP4 protein by immunoblot. **C)** SBI-3m recognized overexpressed mouse LTBP4 protein, including both the 129 and D2 forms, after overexpression in HEK cell lysates. UT = untransfected HEK lysates.

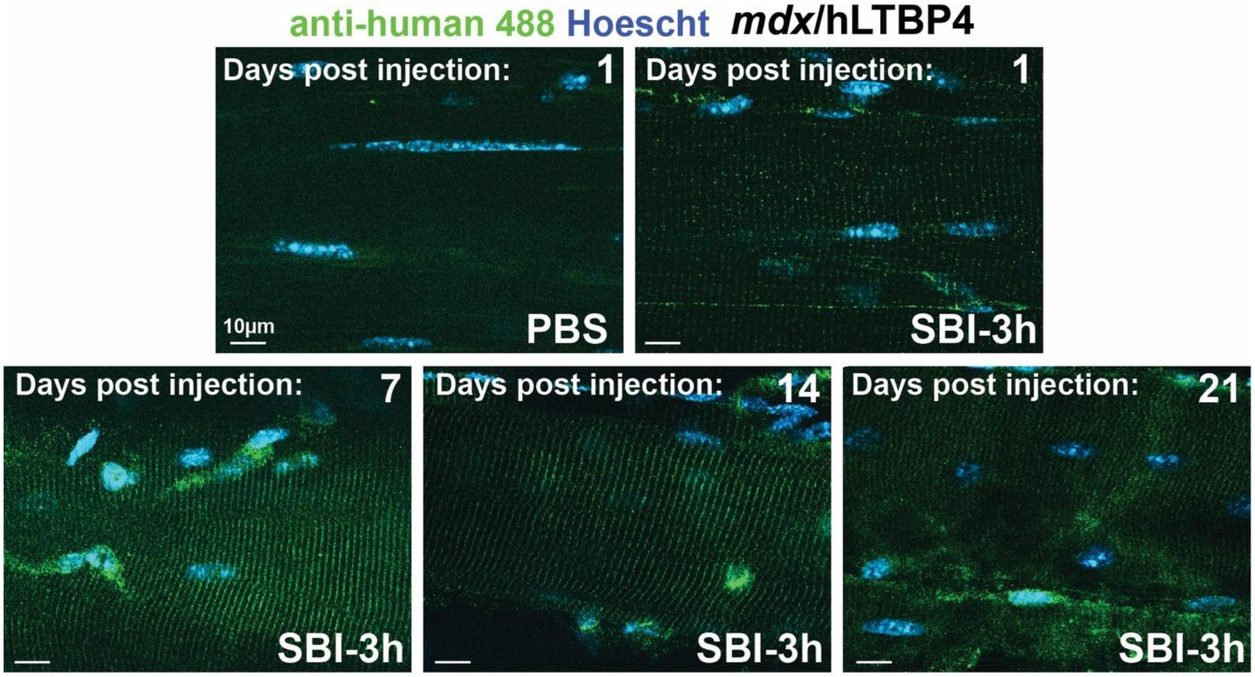

Figure S2. Human anti-LTBP4 monoclonal antibody was detectable at muscle membranes for at least 21 days after a single antibody injection. *mdx*/hLTBP4 male mice were injected i.p. with SBI-3h at 10 mg/kg once and sacrificed 1, 7, 14, or 21 days later. Control mice were injected with PBS once and sacrificed 1 day later. Muscles were harvested and probed with anti-human IgG secondary antibodies. Longitudinal imaging of the extensor digitorum longus (EDL) muscle detected the presence of costameric, anti-human IgG fluorescence signal (green) as much as 21 days after injection of human anti-LTBP4 antibody (SBI-3h), consistent with target engagement. Costameric fluorescence was not detected in PBS-injected control mice. Scale bar 10 µm.

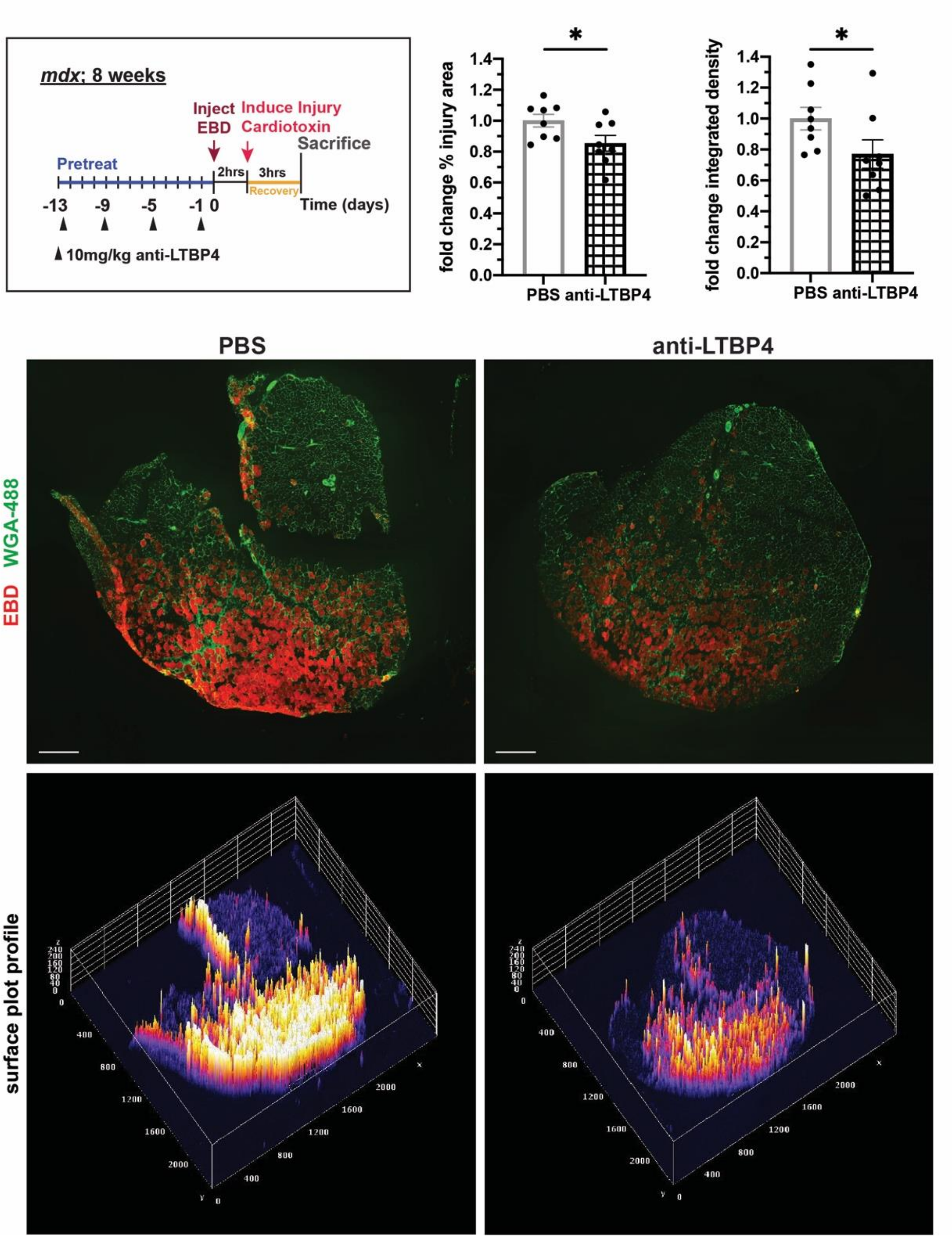

Figure S3. Anti-LTBP4 protects muscle from injury in vivo. *mdx* mice were pretreated with 10 mg/kg anti-LTBP4 antibody or PBS every 4 days for 2 weeks. Mice were then injected with Evan's blue dye (EBD). Two hours after dye injection, the tibialis anterior muscle was injured with cardiotoxin. Muscle was allowed to recover for 3 hours after cardiotoxin injury to capture an early phase of injury response to assess whether LTBP4 antibody protects against injury. Anti-LTBP4 treatment reduced EBD-positive (red) injury area and reduced EBD fluorescence measured as integrated density compared to controls. Representative images and 3-D surface plot profiles are shown. WGA conjugated to 488 (green) was used to outline the muscle. $n \geq 8$ legs per group. *$P<0.05$ by Mann-Whitney test.

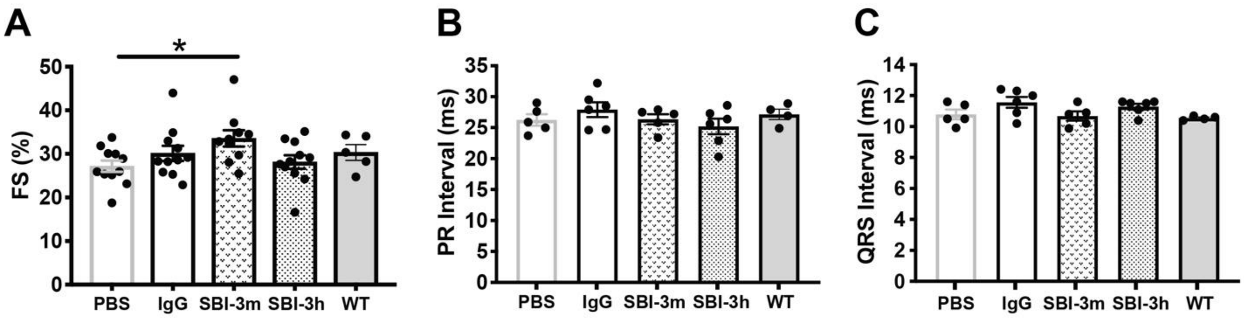

Figure S4. Cardiac outcomes after long-term SBI-3h antibody treatment. *mdx/*hLTBP4 male mice were injected i.p. with either PBS, human IgG control, SBI-3m or SBI-3h at 10mg/kg once per week for 24-weeks. **A)** Cardiac fractional shortening (FS%) was increased in mice treated with SBI-3m compared to PBS controls. **B-C)** No significant changes in PR or QRS intervals were noted between any treatment groups. * $P<0.05$ by one-way ANOVA. (panel A & B, n = 11; panels C & D $n \geq 4$) mice per group.

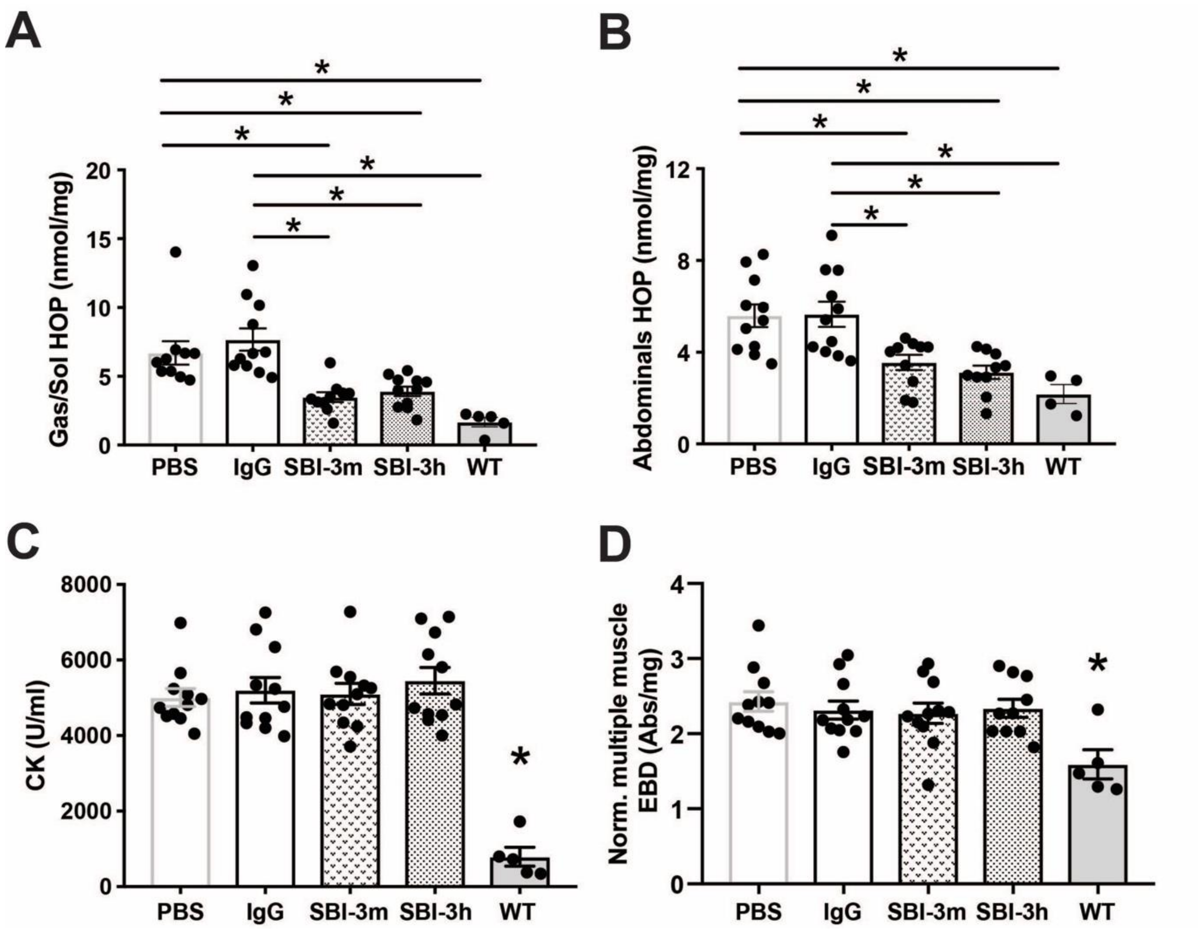

Figure S5. Serum creatine kinase and Evans blue dye uptake were unchanged after long-term anti-LTBP4 treatment. *mdx/*hLTBP4 male mice were injected i.p. with either PBS, human IgG control, SBI-3m or SBI-3h at 10mg/kg once weekly for 24-weeks starting at 8 weeks of age. The last injection was 7 days prior to serum and muscle collection. **A** and **B)** Hydroxyproline content was measured in multiple muscle groups including the gastrocnemius/soleus (Gas/Sol) (A) and abdominal muscles (B). **C)** Serum creatine kinase (CK) after 24 weeks among the groups. **D)** Evan's blue dye (EBD) uptake in multiple muscle groups after 24 weeks of treatment. * $P<0.05$ by one-way ANOVA; WT to all treatment groups. $n \geq 10$ mice per group. WT $n \geq 4$ mice.

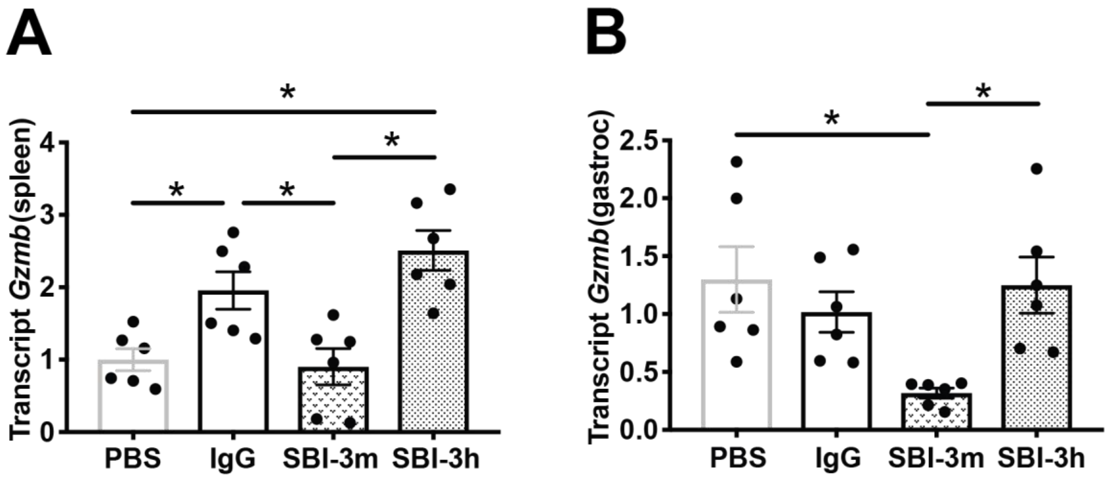

Figure S6 Decreased *Gzmb* expression with long-term anti-LTBP4 mouse antibody treatment. *Gzmb* encodes granzyme B, a marker in cytotoxic T cells and NK cells. Gzmb transcript levels were monitored in the spleen (A) and muscle (B) of *mdx/*hLTBP4 male mice treated for 24 weeks with either PBS, human IgG control, SBI-3m, or SBI-3h. G*zmb* transcript levels were elevated in spleens from mice treated with SBI-3h, the human antibody, and human IgG, potentially consistent with an immune response to the human antibody. The *Gzmb* transcript levels in gastrocnemius muscle treated with SBI-3m was consistent with reduced inflammatory infiltrate in treated dystrophic muscle. * P<0.05 by one-way ANOVA. n = 6 mice per group.

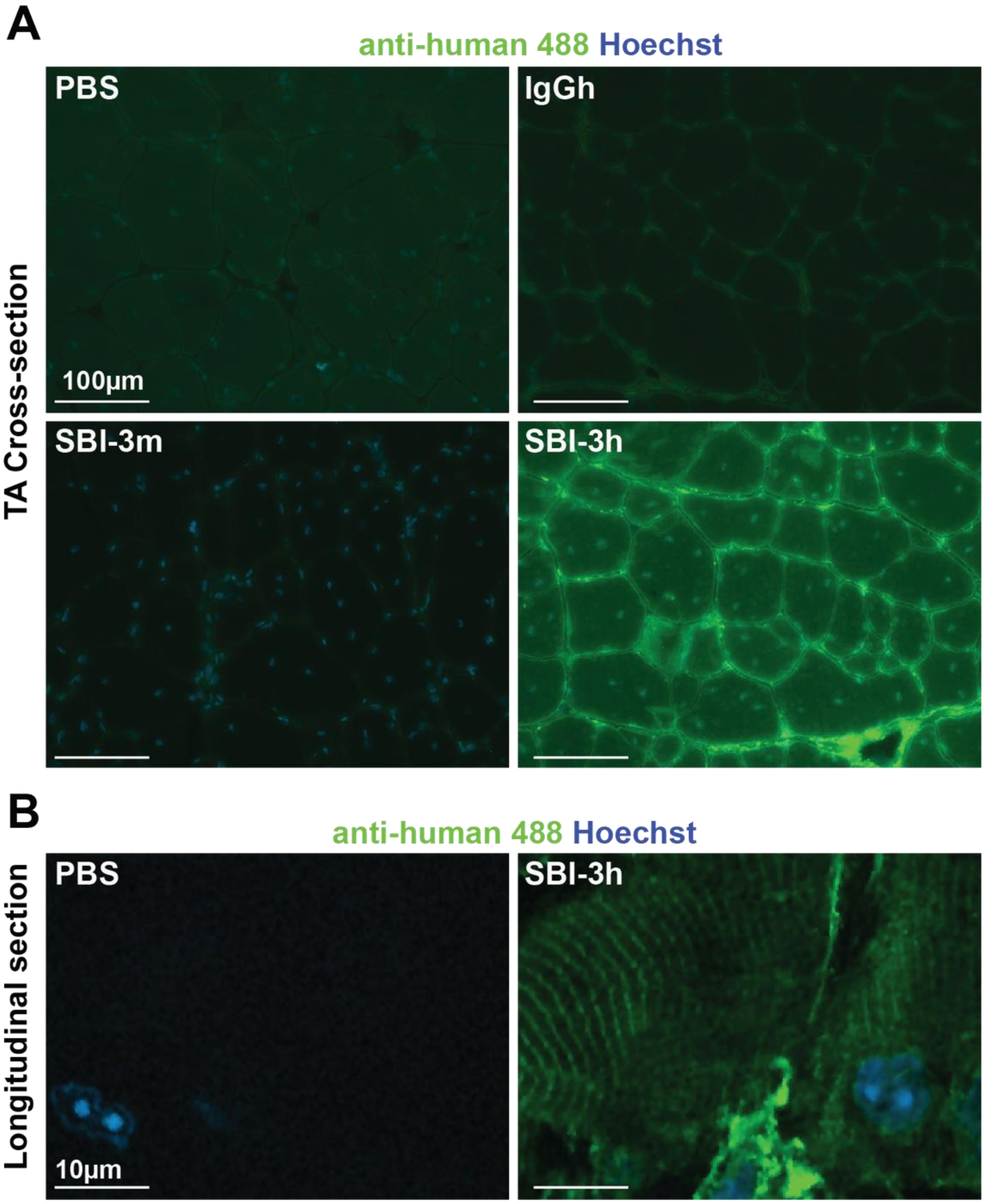

Figure S7. Anti-human antibody detected in muscle from long-term, human anti-LTBP4 treated mice. *mdx*/hLTBP4 male mice were injected i.p. with either PBS, human IgG control, SBI-3m, or SBI-3h antibody at 10mg/kg once per week for 24-weeks starting at 8 weeks of age. The last antibody injection was 7 days prior to muscle harvest. **A)** Anti-human IgG staining (green) detected the presence of human anti-LTBP4 antibody in TA muscle of SBI-3h injected mice, but not in SBI-3m, IgG, or PBS-injected control mice. Scale bar 100 µm. **B)** Longitudinal imaging illustrates anti-human staining in a striated, costameric pattern in mice injected with human anti-LTBP4, but not in PBS control muscle. Hoechst (blue) was used to mark nuclei. Scale bar 10 µm.

Table S1. Summary of outcome measures of *mdx*/hLTBP4 mice treated for 24-weeks with anti-LTBP4 antibody compared to controls.

	PBS	IgG	SBI-3m	SBI-3h
**# mice**	12	12	12	12
**Starting age (D)**	56.8 ± 5.8	56.8 ± 5.8	57.0 ± 6.8	56.8 ± 6.4
**Starting body mass (g)**	26.26 ± 1.9	26.04 ± 2.4	25.99 ± 2.4	26.35 ± 2.0
**# deceased / cause**	1 / puncture	1 / puncture	1 / puncture	1 / puncture
**anti-human IgG muscle IFM detection**	no signal	minimal	no signal	**strong**
**Body mass/tibia (g/mm)**	1.39 ± 0.6	1.42 ± 0.1	1.42 ± 0.06	1.48 ± 0.1
**G/H weight (mg)**	25.00 ± 2.3	25.39 ± 3.8	24.77± 3.4	**27.75± 3.1***
**CSA (μm^2)**	889 ± 89.6	903 ± 72.8	**1049 ± 107.3***	**1044 ± 98.8***
**Tetanic Force (mN)**	241.8 ± 48.5	235.3 ± 55.8	**378 ± 32.4***	**379.5 ± 42.8***
**Specific Force (mN/μm^2)**	0.27 ± 0.06	0.26 ± 0.08	**0.36 ± 0.05***	**0.37 ± 0.06***
**Decreased Contraction Time (ms)**	242.7 ± 15.8	245.4 ± 16.9	**219.9 ± 11.4***	**217.9 ± 14.2***
**Cardiac FS (%)**	27.17 ± 4.3	30.1 ± 5.7	**33.6 ± 5.9***	28.2 ± 5.1
**HOP Diaphragm (nmol/mg)**	19.2 ± 4.4	17.4 ± 4.1	**10.7 ± 3.5***	**8.6 ± 2.9***
**HOP gas/sol (nmol/mg)**	6.7 ± 2.7	7.7 ± 2.7	**3.48 ± 1.1***	**3.9 ± 1.2***
**WBP Penh/mass (AU)**	0.055 ± 0.004	0.067 ± 0.002	**0.04 ± 0.004 ***	**0.046 ± 0.002** *
**pSMAD 1/3/5 Fluor (AU)**	3.8 ± 2.0	**1.5 ± 0.5***	**1.5 ± 1.1***	**0.9 ± 0.4***
**# internal nuclei / myofiber**	1.4 ± 0.25	1.3 ± 0.17	1.5 ± 0.45	1.3 ± 0.26
**QRS interval (ms)**	10.8 ± 0.70	11.6 ± 0.86	10.7 ± 0.66	11.3 ± 0.47
**Grip strength (N/g)**	0.037 ± 0.007	0.038 ± 0.007	0.036 ± 0.005	0.035 ± 0.003
**Multiple muscle EBD (abs/mg)**	2.4 ± 0.43	2.3 ± 0.39	2.2 ± 0.45	2.3 ± 0.38
**serum CK (U/ml)**	5009 ± 784	5201 ± 1123	5104 ± 936	5456 ± 1162

*P <0.05 by one-way ANOVA

[D, days; IFM, immunofluorescence microscopy; G/H, gluteus/hamstring; CSA, cross-sectional area; FS, fractional shortening; HOP, hydroxyproline; WBP, whole body plethysmography; EBD, Evans blue dye; CK, creatine kinase; AU, arbitrary units; Fluor, fluorescence intensity]

Data file S1. Raw data.
